# Comparing Geodesic Filtering to State-of-the-Art Algorithms: A Comprehensive Study and CUDA Implementation

**DOI:** 10.3390/jimaging11050167

**Published:** 2025-05-20

**Authors:** Pierre Boulanger, Sadid Bin Hasan

**Affiliations:** Department of Computing Science, University of Alberta, Edmonton, AB T6G2R3, Canada; sadidbin@ualberta.ca

**Keywords:** geodesic filtering, anisotropic diffusion, image processing, PSNR, noise reduction, edge preservation, GPU implementation, manifolds, Riemannian space

## Abstract

This paper presents a comprehensive investigation into advanced image processing using geodesic filtering within a Riemannian manifold framework. We introduce a novel geodesic filtering formulation that uniquely integrates spatial and intensity relationships through minimal path computation, demonstrating significant improvements in edge preservation and noise reduction compared to conventional methods. Our quantitative analysis using peak signal-to-noise ratio (PSNR) and structural similarity index (SSIM) metrics across diverse image types reveals that our approach outperforms traditional techniques in preserving fine details while effectively suppressing both Gaussian and non-Gaussian noise. We developed an automatic parameter optimization methodology that eliminates manual tuning by identifying optimal filtering parameters based on image characteristics. Additionally, we present a highly optimized GPU implementation featuring innovative wave-propagation algorithms and memory access optimization techniques that achieve a 200× speedup, making geodesic filtering practical for real-time applications. Our work bridges the gap between theoretical elegance and computational practicality, establishing geodesic filtering as a superior solution for challenging image processing tasks in fields ranging from medical imaging to remote sensing.

## 1. Introduction

The central challenge in image filtering lies in achieving an optimal balance between noise reduction and the preservation of essential image features. Traditional methods rely on carefully designed mathematical models to selectively smooth images while retaining edges and textures. In contrast, modern neural network approaches employ data-driven learning to obtain similar objectives. However, both methodologies face inherent limitations: conventional methods often struggle with complex noise patterns, whereas neural networks typically require extensive training data and frequently lack robust theoretical guarantees, leading to unpredictable behavior.

In response to these challenges, this paper makes four specific contributions to the field of image processing:

1. Novel Geodesic Filtering Framework: We introduce a mathematically rigorous formulation of geodesic filtering that uniquely leverages Riemannian manifold theory to combine spatial and intensity information in a unified framework. Unlike other filters, the geodesic approach adapts to local image characteristics through
  - Geometric distance metrics in a manifold: Allowing for a more natural measurement of similarity between pixels by integrating both spatial and intensity relationships.
  - Local curvature considerations: Preserving the intrinsic structure of image features, especially along edges and contours.
  - Adaptive kernel sizing based on manifold properties: Dynamically adjusting the filtering window in response to the local geometry of the image.

Unlike previous approaches that treat these domains separately, our method computes minimal geodesic paths that inherently respect image structure, offering significant advantages, including

  - Enhanced edge preservation: Retaining sharp transitions and fine details.
  - Superior noise reduction: Effectively suppressing both Gaussian and non-Gaussian noise.
  - Fine texture detail retention: Maintaining subtle textures while reducing unwanted noise.
  - Effective handling of variable noise levels: Adapting robustly to different noise intensities across the image.
  - Sharp transition preservation during noise smoothing: Avoiding over-smoothing at boundaries.
  - Enhanced performance with non-Gaussian noise: Outperforming traditional methods in challenging noise conditions such as speckle noise.
  - Improved outlier robustness: Providing resilience against anomalous data points.

2. Comprehensive Comparative Analysis: We present the first systematic evaluation of geodesic filtering against state-of-the-art alternatives (including anisotropic diffusion, bilateral filtering, least median of squares, and deep image prior techniques) using standardized metrics (PSNR and SSIM) across diverse image types and noise conditions.
3. Parameter Optimization Framework: We developed a novel methodology for automatically determining optimal filtering parameters based on image characteristics. This approach eliminates the manual trial-and-error process typically required in advanced filtering techniques, making geodesic filtering more accessible for practical applications.
4. High-Performance CUDA Implementation: We introduce specific technical innovations in our GPU implementation that overcome the inherent computational complexity of geodesic filtering. Our wave-propagation algorithm and memory conflict resolution techniques achieve a 200× speedup over conventional CPU implementations, transforming geodesic filtering from a theoretically superior but computationally prohibitive method into a practical solution for real-time applications.

These contributions collectively bridge the gap between theoretical mathematical models and practical image processing applications, establishing geodesic filtering as both a robust theoretical framework and an effective computational tool for advanced image processing.

The remainder of this paper is organized as follows. In Section 2, we review the current literature on non-linear methods for image filtering, detailing their operational principles and implementation strategies. Section 3 introduces the fundamental mathematical concepts underlying geodesic filtering within a Riemannian manifold framework. In Section 4, we present extensive experimental results obtained from processing a diverse image database to demonstrate the impact of various filtering parameters on performance. We also conducted a comparative analysis with other state-of-the-art non-linear filtering techniques. Section 5 describes our highly optimized CUDA C implementation that leverages GPU parallelism to address the computational challenges of geometric filtering. Finally, Section 6 concludes the paper by discussing the strengths and limitations of our approach and Section 7 outlines potential extensions to more complex 3D and 4D manifolds.

## 2. Literature Review

Early methods primarily targeted basic noise reduction using straightforward linear or spatial operations, often at the expense of image structure. Non-linear filtering methodologies represent a sophisticated class of image processing techniques that go beyond traditional linear approaches. These can be categorized into four distinct approaches, each with unique mathematical foundations and operational principles.

Anisotropic diffusion, the first category, draws from partial differential equations and heat flow theory. This approach intelligently modulates the diffusion process based on local image characteristics, allowing homogeneous regions to be smoothed while crucial edge features remain intact. By adapting the diffusion coefficient to the local gradient magnitude, these filters can reduce noise while preserving the structural integrity of images.

The second approach leverages robust statistics to create filters that minimize the influence of edges during smoothing operations. These methods typically replace standard mean calculations with robust estimators like least median square (LMS) that are less sensitive to outliers, effectively treating edge pixels as statistical anomalies. This statistical foundation allows for effective noise reduction without the edge blurring commonly associated with linear filtering techniques, providing superior performance in environments with varying noise distributions.

Geodesic filtering, the third category, represents a more geometrically oriented approach based on differential geometry and manifold theory. This methodology conceptualizes images as high-dimensional manifolds embedded in feature space. By computing geodesic distances, these filters can adaptively process images according to their inherent geometric properties. This approach excels at preserving fine structures and textural details while removing noise.

The fourth category encompasses neural-network-based methods, which leverage data-driven approaches to optimize filtering parameters. Unlike traditional approaches that rely on predefined mathematical models, these methods learn optimal filtering strategies directly from training data. By exposing networks to pairs of noisy and clean images, these systems can discover complex non-linear relationships that effectively separate signal from noise. The resulting filters often demonstrate remarkable adaptability across various noise types and image characteristics, though their performance depends heavily on the quality and diversity of the training data. This analysis specifically examines algorithms that do not require training data, thus excluding conventional neural network methods. For readers interested in deep-learning-based image denoising techniques, reference [1] offers a comprehensive overview. The Deep Image Prior (DIP) network [2] is the only neural network approach considered here, as it uniquely functions without pre-training requirements.

Each of these approaches offers distinct advantages and limitations, with their effectiveness varying according to specific application requirements, computational constraints, and the nature of the image data being processed.

### 2.1. Gradient Anisotropic Diffusion

Gradient anisotropic diffusion (GAD), as introduced by Perona and Malik [3], fundamentally reimagines image processing through the lens of heat diffusion equations. The core mathematical formulation begins with a partial differential diffusion equation:

(1) $$\frac{\partial I}{\partial t}=div\left(g\left(\left|𝛻I\right|\right)𝛻I\right)$$

where $I\left(x,y,t\right)$ represents the image intensity at position $\left(x,y\right)$ and time t, and $g\left(\left|𝛻I\right|\right)$ is the diffusion coefficient. This formulation builds upon the classical heat equation but introduces crucial non-linearity through the spatially varying diffusion coefficient.

The diffusion coefficient, as noted by Weickert et al. [4], plays a pivotal role in controlling the filtering process. It typically takes the form

(2) $$g\left(\left|𝛻I\right|\right)=g\left(s\right)$$

where g(s) is a function that reduces diffusion coefficient at the edges. Common formulations of $g\left(s\right)$, as discussed by [5], include

(3) $$g\left(s\right)=\exp\left(-\frac{s^{2}}{C^{2}}\right)\;\mathrm{or}\;\;g\left(s\right)=\frac{1}{1+s^{2}/C^{2}}$$

where C represents a threshold parameter controlling edge sensitivity.

The theoretical significance of this framework lies in its ability to achieve selective smoothing. As demonstrated by Alvarez et al. [5], the process preserves edges by reducing diffusion across high-gradient regions while promoting smoothing in homogeneous areas. The mathematical analysis by Weickert et al. [4] showed that this approach creates a scale-space representation with important theoretical properties:

- Causality: No spurious details are created with increasing scale.
- Immediate stabilization: Edge enhancement occurs in early iterations.
- Localization: Edges remain stable during the diffusion process.

On the other hand, GAD exhibits significant sensitivity to parameter configuration, with its effectiveness heavily reliant on appropriate selection of diffusion coefficients and iteration counts. The method frequently encounters stability and convergence challenges when parameters are improperly configured, creating substantial difficulty in establishing automated optimal termination criteria for the diffusion process. Additionally, its filtering capabilities show marked deterioration when confronted with impulse noise patterns or image regions containing complex textures, limiting its applicability across diverse image processing scenarios.

### 2.2. Curvature Anisotropic Diffusion Framework

Curvature anisotropic diffusion (CAD) formulation differs fundamentally from the original work of Perona and Malik by incorporating geometric information through the introduction of a mean curvature term. Initially proposed by Alvarez et al. [6] and further developed by Sapiro et al. [7], this framework offers superior preservation of geometric features while effectively reducing noise. The diffusion equation for curvature anisotropic diffusion is

(4) $$\frac{\partial I}{\partial t}=g\left(k\right)\left|𝛻I\right|div\left(\frac{𝛻I}{\left|𝛻I\right|}\;\;\right)\;$$

where

- $I\left(x,y,t\right)$ represents the image intensity at position $\left(x,y\right)$ and time t;
- k denotes the mean curvature of the level sets;
- $g\left(\kappa \right)$ is a decreasing function that controls the diffusion coefficient;
- 𝛻I is the image gradient;
- div represents the divergence operator.

For the right conditions, the CAD framework demonstrates exceptional ability to preserve critical image features such as edges and boundaries while effectively reducing noise. Its incorporation of geometric curvature information enables superior structural integrity maintenance compared to gradient-based methods. Unlike GAD, CAD incorporates mean curvature of level sets, allowing it to more faithfully respect the intrinsic geometry of image content, particularly along curved structures.

However, CAD’s performance is heavily influenced by appropriate parameter selection. The threshold parameters within the g(κ) function demand careful calibration to achieve optimal results. This makes proper CAD implementation challenging, especially in discrete domains, potentially leading to numerical instabilities. Determining the ideal iteration count presents another non-trivial challenge, frequently requiring manual adjustment or sophisticated stopping criteria. While CAD performs well against Gaussian noise, it may exhibit reduced effectiveness when confronting other noise varieties such as impulse or speckle noise without specific adaptations.

### 2.3. Bilateral Filter Framework

First introduced by Tomasi et al. [8], this non-linear technique revolutionized image processing by combining domain and range filtering in a single, unified framework without having to compute gradients. The method’s fundamental innovation lies in its ability to consider both spatial proximity and photometric similarity simultaneously.

The bilateral filter, as elaborated by Durand and Dorsey [9], operates on two fundamental principles:

- Spatial Domain Filtering: Pixels are weighted based on their spatial distance from the center pixel, following a Gaussian distribution. This component ensures that nearby pixels have more influence than distant ones.
- Signal Domain Filtering: Pixels are additionally weighted based on their photometric (intensity or color) similarity to the center pixel, again using a Gaussian distribution. This component ensures edge preservation by reducing the influence of pixels with significantly different intensities.

Paris et al. [10] formalized its mathematical formulation as

(5) $$I\left(p\right)=\frac{1}{W_{p}}\sum_{q\epsilon \Omega }G_{\sigma_{s}}(\left\|p-q\right\|)G_{\sigma_{r}}(\left|I\left(p\right)-I\left(q\right)\right|$$

where

- I represent the input image intensity;
- p denotes the current pixel position;
- Ω represents the spatial neighborhood;
- q denotes the neighboring pixel position;
- $G_{\sigma_{s}}$ and $G_{\sigma_{r}}$ are Gaussian functions for spatial and range domains;
- $W_{p}$ is the normalization factor.

Studies by Kaplan et al. [11] showed that this algorithm possesses superior noise reduction capabilities:

- Effective reduction of random noise;
- Preservation of underlying signal structure;
- Minimal introduction of artifacts.

On the other hand, bilateral filtering’s effectiveness is significantly contingent on the appropriate selection of spatial and range parameters, necessitating meticulous calibration for optimal results across various applications. While the filter performs admirably against Gaussian noise, it demonstrates reduced efficacy when confronting alternative noise types such as impulse noise or structured noise patterns. Additionally, the filter can generate intensity shift artifacts in high-gradient regions. This creates edge localization challenges where, in areas with gradual transitions, the filter may not accurately maintain edge positions, potentially causing subtle displacement of boundary locations. Certain parameter configurations can also introduce unwanted piecewise constant regions, resulting in an artificial “staircase” effect in what should be smooth gradient areas.

### 2.4. Robust Least Median of Squares Filtering

While traditional filtering approaches like CAD, GAD, or bilateral filtering can address specific noise types, they often struggle with mixed noise patterns or fail to preserve important image features. Using the least median of squares (LMS) regression methods, introduced by Rousseeuw [12,13], offers a robust framework capable of handling up to 50% outlier contamination, making it ideal for edge preserving filters. Recent applications of LMS include edge-preserving smoothing [14] and feature detection [15].

The LMS filter processes images by looking at small windows of pixels of size s×s (typically 5 × 5 or 7 × 7) around a central pixel $\left(x_{0},y_{0}\right)$. Let us define a local image color approximation for this local window as polynomial of degree d:

(6) $$f^{c}\left(x,y;\beta^{c}\right)=\sum_{k=0,l=0}^{k+l\leq d}\beta_{kl}^{c}{\left(x-x_{0}\right)}^{k}{\left(y-y_{0}\right)}^{l}$$

where $\left(x,y\right)$ are the local coordinates of the pixels that are located inside the window; $\beta^{c}=\left[\beta_{kl}^{c}\right]$ is the polynomial coefficients for the color channel c; and d is the order of the polynomial, typically 1 or 2.

The role of an LMS estimator is to determine the best coefficients ${\hat{\beta }}_{kl}^{c}$ that minimize the median error between the pixels in the window and the polynomial model:

(7) $$\underset{\beta^{c}}{\mathrm{argmin}}\underset{i\in s^{2}}{\mathrm{median}}\left({\left(P_{i}^{c}-f^{c}\left(x_{i},y_{i};\beta^{c}\right)\right)}^{2}\right)$$

where $P_{i}^{c}$ is the color value in channel c for pixel $\left(x_{i},y_{i}\right)$ inside the window, $f^{c}\left(x_{i},y_{i};\beta^{c}\right)$ is the local polynomial model, and $s^{2}$ is the number of pixels in the processing window.

In each window, the algorithm tries to fit the polynomial model to the pixel values using a RANSAC (random sample consensus) algorithm by Fischler and Bolles [16] where a minimum sample set equal to $s_{m}=\frac{\left(d+1\right)\left(d+2\right)}{2}$ is used to compute candidate model coefficients $\beta^{c}$(t). For each sampling iteration t, the algorithm computes the median values $D_{med}^{t}$ of the error between the remaining pixels $P_{i}^{c}\left({\hat{x}}_{i},{\hat{y}}_{i}\right)$ and the current instance of the local polynomial model $f_{t}^{c}\left({\hat{x}}_{i},{\hat{y}}_{i};\beta^{c}\left(t\right)\right)$. The number of random sample iterations is determined by $\frac{m=\ln\left(1-p\right)}{\ln\left(1-{\left(1-\varepsilon \right)}^{s_{m}}\right)}$, which guarantees a confidence level of *p* = 0.99 for an outlier ratio of ε = 50%. After m iterations, the algorithm then chooses the model corresponding to the least median square value $D_{med}^{t_{b}}$ and its corresponding model coefficients $\beta^{c}$($t_{b}$). Using this model, the algorithm diagnoses the pixels inside the window that are inliers vs. outliers by computing the difference $d_{i}^{c}=P_{i}^{c}\left({\hat{x}}_{i},{\hat{y}}_{i}\right)-f_{t}^{c}\left({\hat{x}}_{i},{\hat{y}}_{i};\beta^{c}\left(t\right)\right)$ between the LMS model and the remaining pixel. Following Rousseeuw and Leroy [17], the robust threshold $T_{r}$ to determine if a pixel is an inlier vs. outlier is

(8) $$T_{r}=1.4826\times median\left(abs\left(d_{i}-D_{med}^{\prime }\right)\right)$$

One can then diagnose the pixel using the following test: *if* $abs\left(d_{i}\right)\leq 2.5\;T_{r}$ then it is an inlier. Using the inlier pixels, the algorithm then computes the final model coefficients ${\hat{\beta }}^{c}$ using a least mean square algorithm and then replaces the central pixels with the polynomial approximation $f^{c}\left(x_{0},y_{0};{\hat{\beta }}^{c}\right)$. If the number of inliers is smaller or equal to $s_{m}$, then the central pixel is replaced by the median value of the window. For color images, we process each channel independently while maintaining color consistency through

- Joint outlier detection across channels.
- Consistent polynomial surface fitting.
- Color-aware scale estimation.

The LMS approach demonstrates remarkable resilience, handling contamination of up to 50% outliers, which makes it exceptionally robust for edge preservation and noise reduction compared to conventional filtering methods. LMS particularly excels at maintaining crisp edges and boundaries while efficiently eliminating noise, avoiding the blurring artifacts typically associated with alternative filtering techniques. The algorithm exhibits strong performance across diverse noise distributions and can effectively manage mixed noise patterns that frequently challenge other filtering approaches.

However, LMS filtering presents significant challenges in parameter optimization due to the complex interactions between polynomial degree, window size, and outlier threshold parameters that substantially influence performance outcomes. Additionally, LMS demands considerable computational resources, requiring multiple sampling iterations for each processing window, which can significantly impact processing time for larger images or real-time applications.

### 2.5. Deep Image Prior (DIP) Neural Network Filters

Deep image prior (DIP) represents a novel approach that harnesses the inherent structure of convolutional neural networks (CNNs) as an effective regularizer for natural image processing, without requiring pre-training on image datasets. The key insight is that architecture CNN inherently captures image statistics that make it biased toward natural images over noise. When optimizing an untrained CNN to reconstruct a corrupted image by minimizing the reconstruction loss, the network tends to learn the natural image content before fitting noise or artifacts. This approach has proven effective for various image restoration tasks including denoising [2], super-resolution [18], and inpainting [19], all without requiring any training data beyond the single corrupted image being processed. DIP offers several significant advantages over standard neural network algorithms: it enables zero-shot learning through the network’s architecture serving as a natural image prior, provides flexibility across multiple restoration tasks without dataset bias, and offers interpretability in how it captures image statistics. However, DIP faces notable limitations: it is computationally intensive, requiring thousands of iterations per image. The method is also sensitive to early stopping criteria and hyperparameter selection. It also lacks theoretical guarantees relying instead on empirical observations resulting in inconsistent results due to random initialization and dynamics.

One variant of DIP is Wavelet-DIP, which has been shown by Yang, Y., et al. [20] and Liu, C., et al. [21] to enhance the original DIP framework by incorporating wavelet decomposition into the network architecture, leveraging the multi-scale analysis capabilities of wavelets. To improve processing images with Gaussian noise, a similar version to Wavelet-DIP was proposed. Ulyanov et al. introduced a change to Wavelet-DIP called the Gaussian Weighted Wavelet-DIP [22] (GW-DIP) where the wavelet function is first convolved by a Gaussian filter. GW-DIP solves the optimization problem:

(9) $${min}_{\theta }\left\{L\left(f_{\theta }\left(z\right),\;I\right)+\lambda R\left(f_{\theta }\left(z\right)\right)\right\}$$

where $f_{\theta }$ is the neural network with parameters θ, z is a random noise input, x is the target image, R is the regularization term, and λ is a regularization weight. This minimizes the distance between the network output $f_{\theta }\left(z\right)$ and target image I, with regularization R.

Initial random noise $z\;\epsilon \;N\left(0,1\right)$ is transformed through Gaussian weighting:

(10) $$z^{\prime }\left(p\right)=\frac{1}{K\left(p\right)}\sum_{q\;\epsilon \;\Omega }G_{\sigma_{w}}\left(p-q\right)z\left(q\right)\;$$

where K(p) = $\sum_{q\;\epsilon \;\Omega }G_{\sigma_{w}}\left(p-q\right)$ is a normalization factor, $G_{\sigma_{w}}\left(p\right)$ is 2D Gaussian kernel, Ω is spatial neighborhood window, and $p=\left(x_{p},\;y_{p}\right)$ and $q=\left(x_{q},\;y_{q}\right)$ are pixel coordinates.

**Figure 1:**
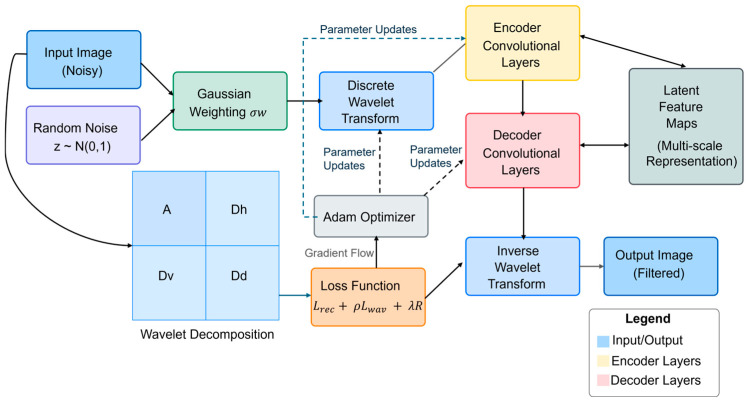
Gaussian Wavelet-DIP architecture.

The feature map F is a discrete wavelet transform (DWT) defined as

(11) $${\{A}_{i,k},\;D_{i,k}^{h},D_{i,k}^{v},D_{i,k}^{d}\}=DWT\left(F\right)$$

where i is the decomposition level, k is the spatial location, $A_{i,k}$ is the low-pass approximation, $D_{i,k}^{h}$ is the horizontal components, $D_{i,k}^{v}$ is the vertical components, and $D_{i,k}^{d}$ is the diagonal components. In our test implementation, we used the Haar wavelet for its simplicity and computational efficiency. One can see in Figure 1 the architecture of the network.

The combined loss function is $L_{total}=L_{rec}+\rho L_{wav}+\lambda R\left(f_{\theta }\left(z\right)\right)$ where $L_{rec}={\left|f_{\theta }\left(z\right)-I\right|}^{2}$ is the reconstruction loss function, $L_{wav}=\sum_{i,k}{\left|DWT\left(f_{\theta }\left(z\right)\right)\left(i,k\right)-DWT\left(I\right)\left(i,k\right)\right|}^{2}$ is the wavelet loss function, and $R\left(f_{\theta }\left(z\right)\right)=\sum_{p}\left|\nabla f_{\theta }\left(z\right)\right|$ the total variation regularization term. The parameters θ are updated using Adam optimizer:

(12) $$\theta \left(t+1\right)=\theta \left(t\right)-\pi \times \left(\frac{\hat{m}\left(t\right)}{\sqrt{\hat{v}\left(t\right)}}\right)+\epsilon \;$$

where

- $m\left(t\right)=\beta_{1}m\left(t-1\right)+\left(1-\beta_{1}\right){𝛻}_{\theta }L_{total}$;
- $v\left(t\right)=\beta_{2}v\left(t-1\right)+\left(1-\beta_{2}\right){𝛻}_{\theta }L_{total}^{2}$;
- $\hat{m}\left(t\right)=\frac{m\left(t\right)}{1-\beta_{1}^{t}}$;
- $\hat{v}\left(t\right)=\frac{v\left(t\right)}{1-\beta_{2}^{t}}$.

with typical values of learning rate π=0.001, $\beta_{1}=0.9$, $\beta_{2}=0.99$, and $\epsilon =10^{-8}$.

By processing different frequency components separately through dedicated network branches, GW-DIP can better handle various image features at different scales. The wavelet transform naturally separates an image into low-frequency approximation coefficients and high-frequency detail coefficients, allowing the network to learn appropriate representations for each frequency band. This multi-scale wavelet structure provides additional information that aligns with natural image statistics, as wavelets are known to produce sparse representations of natural images.

GW-DIP leverages the intrinsic structure of convolutional neural networks (CNNs) as an implicit regularization mechanism for natural image processing, eliminating the need for pre-trained data. This method demonstrates remarkable capability in eliminating complex noise patterns that typically challenge conventional filtering techniques, as it adapts to image-specific characteristics. The architectural design of the network inherently preserves significant edges and details while effectively removing noise.

However, DIP demands substantial computational resources, requiring thousands of optimization iterations for processing a single image. The approach necessitates vigilant monitoring and strategic early stopping to prevent the network from eventually fitting to noise patterns, which complicates automation efforts. Furthermore, the quality of results significantly depends on several factors including network architecture, learning rate, and various hyperparameters that require meticulous tuning for optimal performance.

## 3. Geodesic Filtering

Geodesic filtering, first introduced by Boulanger [23] to process range data $x\left(u,v\right),\;y\left(u,v\right),\;z\left(u,v\right)$ and later by Sochen et al. [24] to process intensity images, addresses image filtering from a fundamentally different mathematical perspective. To generalize this work, we introduce a novel filtering framework that treats signals as a m-dimensional Riemannian manifold Π embedded in a n-dimensional Euclidian space, typically combining spatial and signal value coordinates. Let $r\left(\Pi_{p}\right)$ be a n-dimensional vector immersed into a m-dimensional rectangular manifold with coordinate $\Pi_{p}$. For 2D images, the manifold coordinates of a point p is $\Pi_{p}=\left(u_{p},v_{p}\right)$, corresponding to a mapping from $R^{2}$ to $R^{n}$ such as:

(13) $$\Pi_{p}\to \left(u_{p},v_{p}\right)\to \;\left(x\left(u_{p},v_{p}\right),y\left(u_{p},v_{p}\right),r\left(u_{p},v_{p}\right)\right)$$

In the case of a color image, the mapping is $R^{2}$ to $R^{5}$, which is a mapping from $\left(u,v\right)$ to $\left(x\left(u,v\right),y\left(u,v\right),R\left(u,v\right),G\left(u,v\right),B\left(u,v\right)\right)$ and for grey-level images $R^{2}$ to $R^{3}$ a mapping from $\left(u,v\right)$ to $\left(x\left(u,v\right),y\left(u,v\right),I\left(u,v\right)\right)$.

The geodesic distance between two points $p\left(\Pi \right)$ and $q\left(\Pi \right)$ on Π is defined as

(14) $$d\left(p\left(\Pi \right),q\left(\Pi \right)\right)={inf}_{\gamma }\sqrt{d{x\left(\Pi \right)}^{2}+d{y\left(\Pi \right)}^{2}+\alpha^{2}{\left\|dr\left(\Pi \right)\right\|}^{2}}$$

where ${inf}_{\gamma }$ function is taken over all possible shortest paths γ connecting $p\left(\Pi \right)$ and $q\left(\Pi \right)$ on the manifold. The parameter α weights the importance between the spatial components and the signal differences. This formulation, as analyzed by Kimmel et al. [25], naturally incorporates both spatial and signal value differences into a single geometric framework. The advantages of using geodesic distance on a Riemannian manifold are

- Geodesic distance provides intrinsic measure of similarity;
- Accounts for both spatial and signal differences;
- Preserves image structure better than Euclidean metrics;
- Adapts to local image geometry.

The filtering process utilizes geodesic distances through a weighted averaging:

(15) $$r^{\prime }\left(p\left(\Pi \right)\right)=\frac{\int w\left(\Pi \right)r\left(q\left(\Pi \right)\right)dq}{\int w\left(\Pi \right)dq}$$

where $w\left(\Pi \right)$ is a decreasing function of the geodesic distance relative to $p\left(\Pi \right)$, such as

(16) $$w\left(\Pi \right)=\exp\left(-\frac{d{\left(p\left(\Pi \right),q\left(\Pi \right)\right)}^{2}}{2\sigma^{2}}\right)$$

The theoretical properties of this framework, as established by Boulanger [24] and later by Mémoli and Sapiro [26], include

- Intrinsic geometry preservation;
- Adaptive neighborhood consideration;
- Natural handling of curved structures;
- Topology preservation.

The geodesic distance between points on this manifold incorporates both spatial and signal/geometry differences, providing a natural mechanism for edge-preserving smoothing. This distance measure, fundamental to the filtering process, respects the intrinsic structure of the image rather than relying solely on Euclidean distances in ambient space, making the filtering invariant to rigid transformation for range data.

Peyré [27] further developed these concepts, introducing efficient computational schemes and establishing important theoretical properties of the filtering process. The framework demonstrates several advantageous properties, including rotation invariance, contrast invariance, and the preservation of significant image features.

Modern implementations of geodesic filtering incorporate several sophisticated features. Castaño-Moraga et al. [28] introduced tensor-based extensions that better handle directional features and complex textures. Their work demonstrated improved performance in preserving fine details while still effectively reducing noise.

Zhang et al. [29], develops geometric filtering and edge detection algorithms for non-Euclidean image data, viewing image data as residing on a Riemannian manifold. They extend classical filtering techniques like median filtering and Perona-Malik anisotropic diffusion to handle non-Euclidean data through geodesic distances and the exponential map.

### 3.1. Geodesic Convolution on a Discrete Manifold

Geodesic filtering implementation requires careful consideration of both theoretical principles and practical computational aspects. Implementing geodesic filtering on a discrete manifold follows the theoretical framework established by Boulanger [23] and Sochen et al. [24]. From Equation (15), geodesic convolution is defined on a discrete manifold as

(17) $${\hat{r}}_{\alpha ,\sigma }\left(u_{o},v_{o}\right)=\frac{1}{N\left(u,v\right)}\sum_{u\in w_{u}}\sum_{v\in w_{v}}r\left(u,v\right)e^{-\frac{d^{2}\left(r\left(u,v\right),r\left(u_{o},v_{o}\right),\alpha \right)}{2\sigma^{2}}}$$

where $d\left(r\left(u,v\right),r\left(u_{o},v_{o}\right),\alpha \right)$ is the geodesic distance between $r\left(u,v\right)$ in the window neighborhood of size ($w_{u},w_{v})$ and the center of the window $r\left(u_{o},v_{o}\right)$. $N\left(u,v\right)$ is the normalization factor equal to

(18) $$N\left(u,v\right)=\sum_{u\in w_{u}}\sum_{v\in w_{v}}e^{-\frac{d^{2}\left(r\left(u,v\right),r\left(u_{o},v_{o}\right),\alpha \right)}{2\sigma^{2}}}\;\;$$

Modern implementations incorporate adaptive parameter selection schemes as proposed by Alonso-González et al. [30]. In our implementation the parameters to be adjusted are:

- *α* (signal weighting) based on local gradient statistics;
- σ (filtering extent) based on local noise estimates;
- Window size W based on feature scale analysis.

### 3.2. Minimal Patch Calculation on a Discrete Convolution Window

The computation of minimal paths within local windows is a critical component of geodesic filtering, as it directly influences how image features affect the overall filtering process. Initially formalized by Boulanger [23] and Sethian [31], this process determines the optimal paths that capture the intrinsic geometry of an image. The accuracy and efficiency of these minimal path calculations not only dictate the quality of the filtered output but also have a significant impact on the algorithm’s computational performance. Over the years, various methods have been developed and evaluated for this task. In this work, we implement two primary algorithms: (a) Dijkstra’s algorithm, optimized for scalar processors, and (b) the fast-marching method, which is tailored for efficient GPU-based parallel implementation.

#### 3.2.1. Dijkstra’s Algorithm

The foundation of minimal path calculation lies in graph theory, with Dijkstra’s algorithm [32] serving as a seminal work in this area. In the context of range image processing, as elaborated by Boulanger [23], the inherently discrete nature of digital images maps naturally onto graph structures, where pixels become vertices, and their relationships are represented by weighted edges. Kimmel et al. [25] further advanced this concept by developing continuous formulations that bridge the gap between discrete and continuous manifolds, thereby reinforcing the theoretical underpinnings of geodesic filtering. Extensive analysis by Sethian et al. [31] has shown that under suitable conditions, discrete graph-based methods converge with the solutions obtained from continuous manifold formulations. This theoretical bridge, further refined by Mirebeau [33], forms the basis for modern hybrid approaches that blend discrete and continuous perspectives.

Furthermore, the work of Mémoli and Sapiro [26] established several essential theoretical properties for minimal path computations that any robust method must satisfy:

- Consistency of discrete approximations: Ensuring that as the discretization is refined, the calculated paths converge to the continuous geodesics.
- Convergence rates under refinement: Providing guarantees on the speed and accuracy with which the discrete solution approximates the continuous solution.
- Stability with respect to perturbations: Maintaining reliable performance even when the input data is subject to noise or other perturbations.

By adhering to these foundational principles, our implementation of Dijkstra’s algorithm achieves both high accuracy and computational efficiency, serving as a robust baseline for minimal path calculation in geodesic filtering.

#### 3.2.2. Dijkstra’s Algorithm Implementation

**Figure 2:**
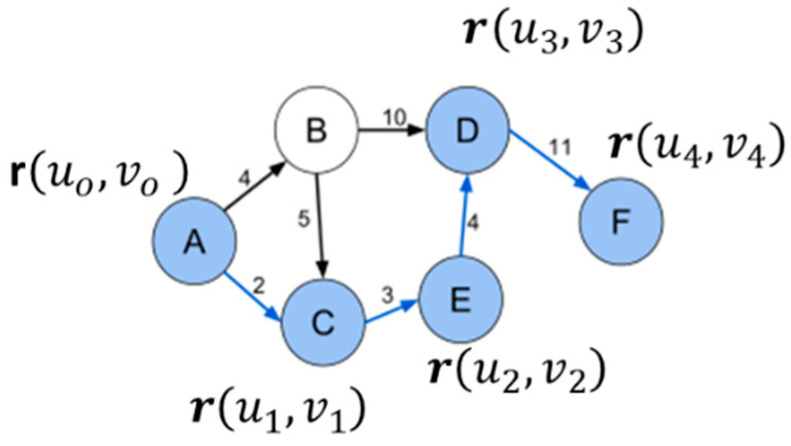
Example of a single-source (vertex A) multiple destination graph (vertices B,C,D,E,F).

The conversion of image data to graph structure, as formalized by Boulanger [23] and Vincent [34], requires careful consideration of both spatial and signal relationships. Let $G=\left(V,E\right)$ be a graph representation of an image where (see Figure 2)

- Vertex set V represents pixel locations;
- Edge set E connects neighboring pixels;
- Weight function w: E → R+ incorporates distance metrics.

The fundamental mappings are

1. Each pixel corresponds to a vertex in the graph, with edges connecting neighboring pixels. The connectivity pattern, typically 4-connected or 8-connected, significantly influences path calculation accuracy. Kimmel et al. [26] demonstrated that 8-connectivity provides better angular resolution at the cost of increased computational complexity.
2. The edge weights incorporate both spatial and signal value information. The general form of edge weight between pixels p and q is defined by Equation (14).
3. The efficiency of priority queue operations becomes crucial in image processing applications. Recent work by Lewis [35] demonstrated that while Fibonacci heaps offer optimal theoretical complexity, as demonstrated in Boulanger [23], binary heaps often perform better in practice due to simpler operations.

Our implementation begins with the definition of essential data structures. We define a pixel structure that contains the following components: spatial coordinates $\left(x\left(u,v\right),y\left(u,v\right)\right)$, signal value $r\left(u,v\right)$, current computed distance from source, a visited flag, and a predecessor reference for path reconstruction. Additionally, an edge structure is defined to represent connections between pixels, containing references to start and end pixels along with a weight value that combines spatial and intensity differences.

**Figure 3:**
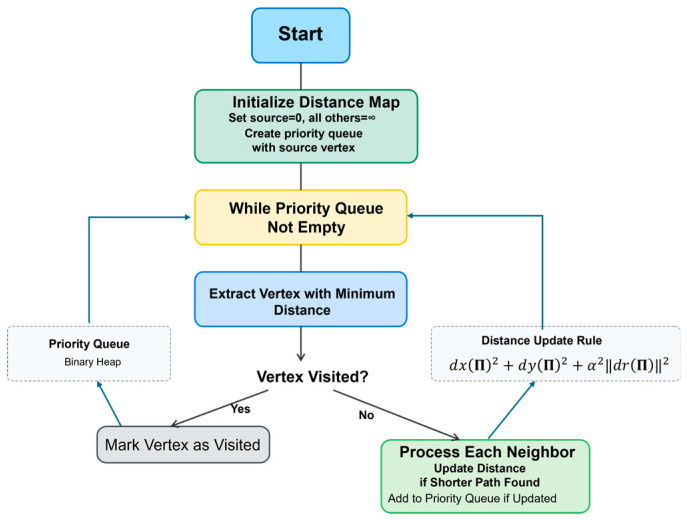
Dijkstra’s algorithm block diagram.

A priority queue structure is implemented using a binary heap, as recommended by Boulanger [23] maintaining pairs of distance values and pixel references. The queue supports three primary operations: insertion of new elements, extraction of minimum-distance elements, and key decrease operations for distance updates. The algorithm works as follows (see Figure 3):

1. Initialization Process

We create a two-dimensional array (pixel_grid) representing the image dimensions. For each position in the image, we instantiate a pixel object with coordinates matching its position, signal values from the original image, distance initialized to infinity (except for the source pixel which gets zero), visited flag set to false, and null predecessor reference. The source pixel’s distance is set to zero, and it is inserted into the priority queue with this initial distance. This setup establishes the starting point for the algorithm’s propagation phase.

2. Propagation Phase

The core processing loop operates as follows. While the priority queue is not empty, we are repeatedly

  - Extracting the pixel with minimum distance from the queue;
  - If the pixel has already been visited, skip to next iteration;
  - Mark the current pixel as visited;
  - Process all neighbors of the current pixel.

For each unvisited neighbor, we

1. Calculate a new potential distance combining
  - Spatial distance between pixels;
  - Signal difference magnitude weighted by parameter β.
2. If the new distance is smaller than the neighbor’s current distance,
  - Update the neighbor’s distance;
  - Set the current pixel as the neighbor’s predecessor;
  - Insert the neighbor into the priority queue with its new distance.
3. Neighbor Processing
  The neighbor processing phase implements an 8-connectivity pattern. For the current pixel position $\left(u,v\right)$, we examine all eight adjacent positions:
  - Horizontal neighbors: $\left(u\pm 1,\;v\right)$;
  - Vertical neighbors: $\left(u,\;v\pm 1\right)$;
  - Diagonal neighbors: $\left(u\pm 1,\;v\pm 1\right)$.
  For each potential neighbor position, we
  - Verify position validity within image boundaries;
  - Calculate combined spatial–intensity distance;
  - Process distance updates if necessary.
4. Distance Calculation
  The distance calculation combines spatial and signal components:
  - Calculate the Euclidean distance $d_{S}^{2}\left(u,v\right)$ between neighboring pixel coordinates $\left(u,v\right)$ and ${(u}^{\prime },v^{\prime }):$
    (19) $$d_{S}^{2}\left(u,v\right)=({\left(x\left(u,v\right)-x(u^{\prime },v^{\prime }\right)}^{2}+{\left(y\left(u,v\right)-y(u^{\prime },v^{\prime }\right)}^{2}$$
  - Account for diagonal connections with appropriate scaling;
  - Compute the norm of the n-dimensional signal difference $r\left(u,v\right)$ and $r\left(u^{\prime },v^{\prime }\right):$
    (20) $$d_{r}^{2}\left(u,v\right)={\left\|r\left(u,v\right)-r\left(u^{\prime },v^{\prime }\right)\right\|}^{2}$$
  - Use a parameter α to assess the significance of the signal difference in comparison to the spatial component.
5. *Final Distance*
  - Combine components using square root of sum of squares;
  - Apply any additional feature-based weighting.

#### 3.2.3. Time and Memory Complexity

The time complexity of Dijkstra’s algorithm for an image of size M×N=s:

- $O\left(slog\;s\;\right)$ using binary heap;
- $O\left(s+Elog\;s\;\right)$ for a Fibonacci heap where E is the number of edges.

Even though the algorithm is slog⁡s efficient, because of the random access to the heap memory, the algorithm is highly inefficient from a memory access point-of-view for parallel implementation, which requires coalesced memory access. For this reason, a second algorithm, more amicable to parallel processing, was implemented for the CUDA version.

### 3.3. Emphasizing Structural Integrity in Geodesic Filtering

Structural integrity represents one of the most significant advantages of geodesic filtering over conventional approaches. This aspect deserves particular emphasis as it directly addresses a fundamental challenge in image processing: preserving essential image structures while effectively removing noise.

By modeling images as high-dimensional manifolds and computing distances along the manifold surface rather than in ambient Euclidean space, geodesic filtering inherently respects the intrinsic geometry of image content. This fundamental difference allows it to

1. Preserve Edge Continuity: Unlike bilateral filtering or anisotropic diffusion that can fragment edges under high noise conditions, geodesic filtering maintains continuous edge structures even with significant noise contamination. This is because geodesic paths naturally follow edge contours along the manifold surface.
2. Maintain Topological Properties: The approach preserves important topological relationships between image regions, ensuring that connected components remain connected and boundaries remain intact after filtering. This is crucial for downstream tasks like segmentation or feature extraction.
3. Adapt to Intrinsic Feature Scale: The geodesic distance calculation automatically adapts to the local feature scale, providing stronger preservation of fine details in textured regions while still effectively smoothing homogeneous areas.
4. Respect Perceptual Organization: By following the natural organization of visual information in the image, geodesic filtering produces results that better align with human visual perception, maintaining the hierarchical structure of image content.

Research demonstrates that with optimized parameter configuration, geodesic filtering consistently surpasses alternative methodologies in preserving structural elements across a wide spectrum of image types. This performance differential becomes particularly significant in demanding applications such as medical imaging, where maintaining the structural integrity of anatomical features directly impacts diagnostic reliability. The exceptional structural preservation achieved through geodesic filtering represents not merely an incremental enhancement in visual quality but a fundamental advancement in preserving the semantic significance of visual information throughout the filtering process.

### 3.4. Comparison of Computational Complexity: The Overall Computing Complexity for a M·N Image for Each Method Is

- Geodesic Filtering: $O\left(M\cdot Ns^{2}\;\right)$;
- LMS Filter: $O\left(M\cdot N\times s^{2}\times p\right)$;
- Gradient Anisotropic Diffusion: $O\left(T\cdot M\cdot N\right)$;
- Curvature Anisotropic Diffusion: $O\left(T\cdot M\cdot N\right)$;
- Bilateral Filter: $O\left(M\cdot N\cdot s^{2}\right)$;
- Gaussian Weighted Wavelet DIP: $O\left(T\cdot N\cdot M\cdot log\left(N\cdot M\right)\right)$.

Here, T is the number of iterations, s is the window size, and p = polynomial terms.

## 4. Experimental Results

This section directly addresses our three core contributions through carefully designed experiments. First, we evaluated the theoretical advantages of geodesic distance calculation in preserving image structures. The parameters α and σ are central to our analysis because they represent the fundamental balance between spatial and intensity information in the geodesic framework. Parameter α controls the relative weighting of intensity differences versus spatial proximity, directly influencing edge preservation capabilities. Parameter σ determines the extent of filtering influence, governing the scale at which features are preserved or smoothed. The main goals of our experiments are to systematically explore the parameter space to demonstrate:

1. The existence of optimal parameter combinations that maximize both PSNR and SSIM metrics across diverse image types.
2. The superior performance of geodesic filtering compared to traditional approaches in maintaining edge integrity while reducing noise.
3. The adaptability of the method to different noise conditions without requiring extensive parameter re-adjustments.

### 4.1. Image Dataset

**Figure 4:**
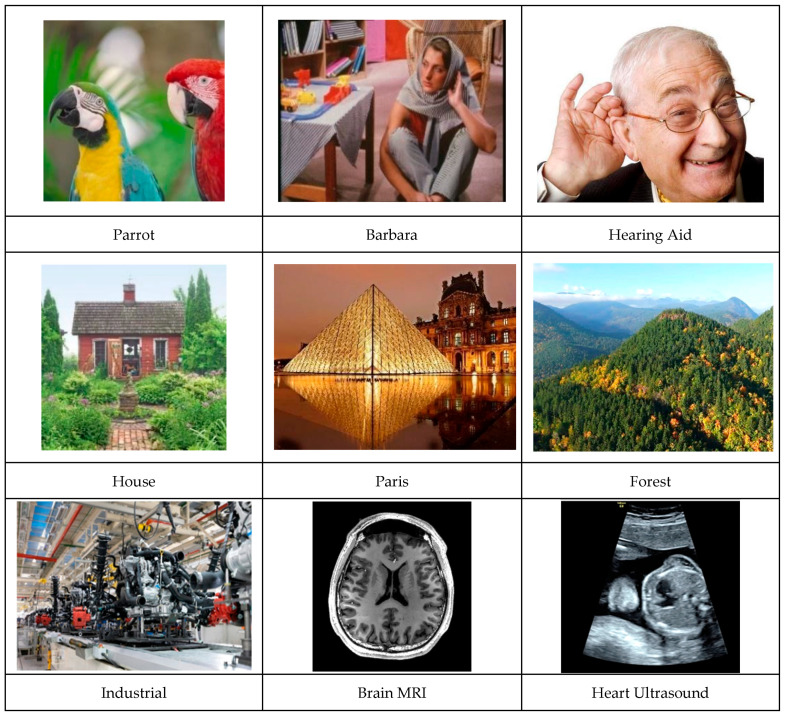
Image database used for the experiments.

To validate the functionality of the algorithms, a standard set of test images was used. These include natural scenes, people, industrial sites, and medical and city images (see Figure 4).

### 4.2. Evolution of Image Dataset vs. Filter Parameters

This section explores the relationship between filter parameters (α and σ) and filtering performance across diverse image types, showing that

1. Each image has an optimal σ value corresponding to its “natural scale”;
2. Parameter α effectively balances spatial and intensity relationships;
3. The filter’s performance peaks at specific parameter combinations, demonstrated through quantitative metrics (PSNR and SSIM).

**Figure 5:**
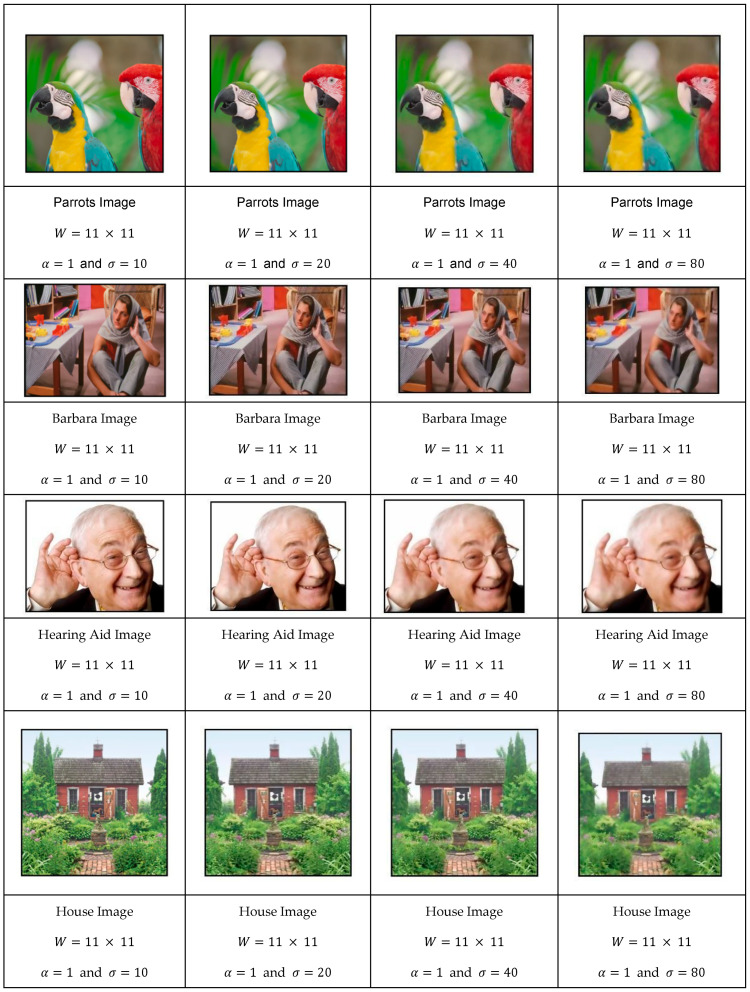

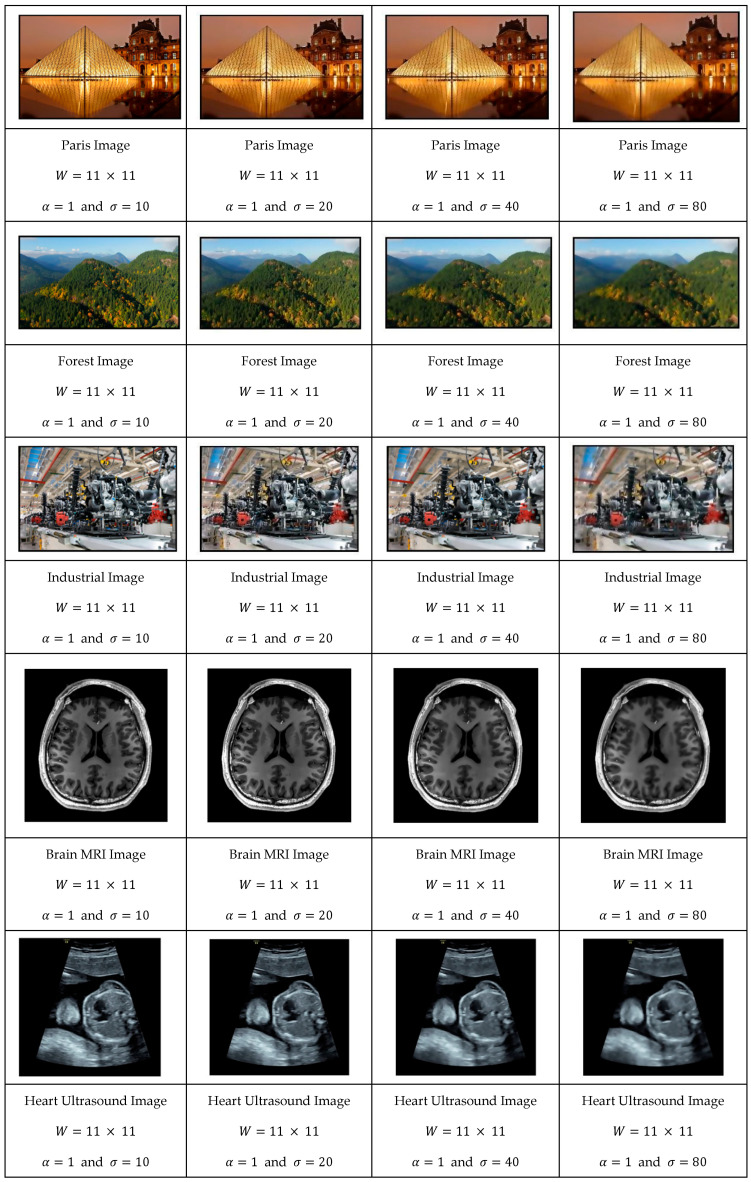
Evolution of the image test database as a function of σ.

In the sequence shown in Figure 5, we convolved the images that were first normalized in size to be of width of 512 pixels and height to a value that respects the aspect ratio of the original image. In these experiments, the convolution window size was set to 11 × 11, and the parameter α equal to 1.

As can be observed, the image’s smoothness level increased proportionally with σ while maintaining edge clarity. As σ values increased, an optimal value σ* emerged, corresponding to an ideal scale. We explore this relationship in greater detail later in our discussion.

Let us now study the effect of the parameter α on the filtering results. One can see the evolution of the filtering process of images (House, Hearing Aid, and Barbara) for various values of α. To highlight its effect, we set the σ to large values (80, 80, and 100).

**Figure 6:**
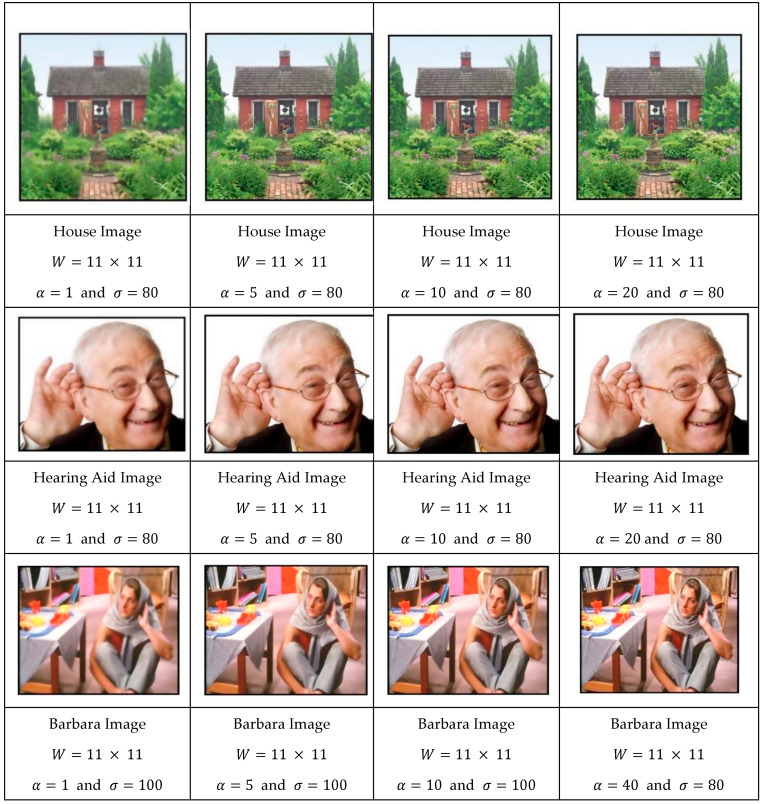
Evolution of the image database as a function of α for a fixed σ.

As one can see in Figure 6, increasing the parameter α reduced the influence of the spatial component, resulting in a filter where pixel value differences became the dominant factor rather than spatial proximity. This shift in dominance led to reduced spatial blurring while still preserving important signal variations across the image.

**Figure 7:**
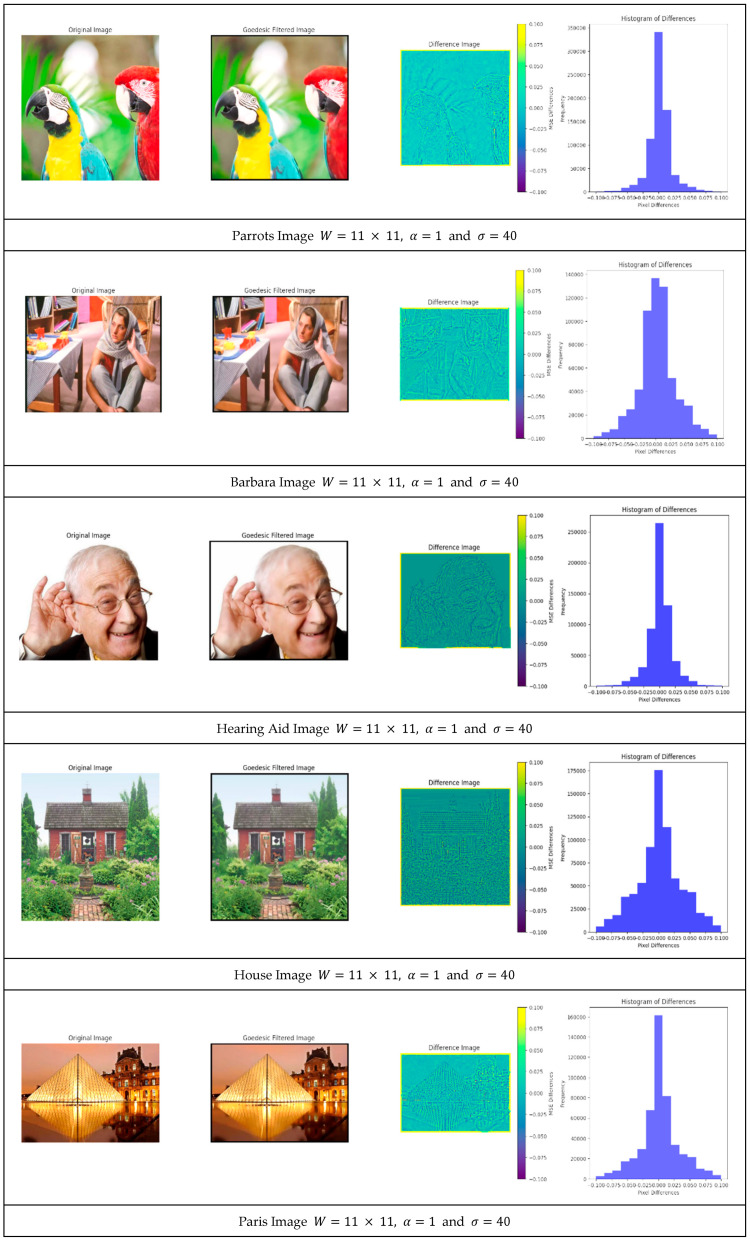

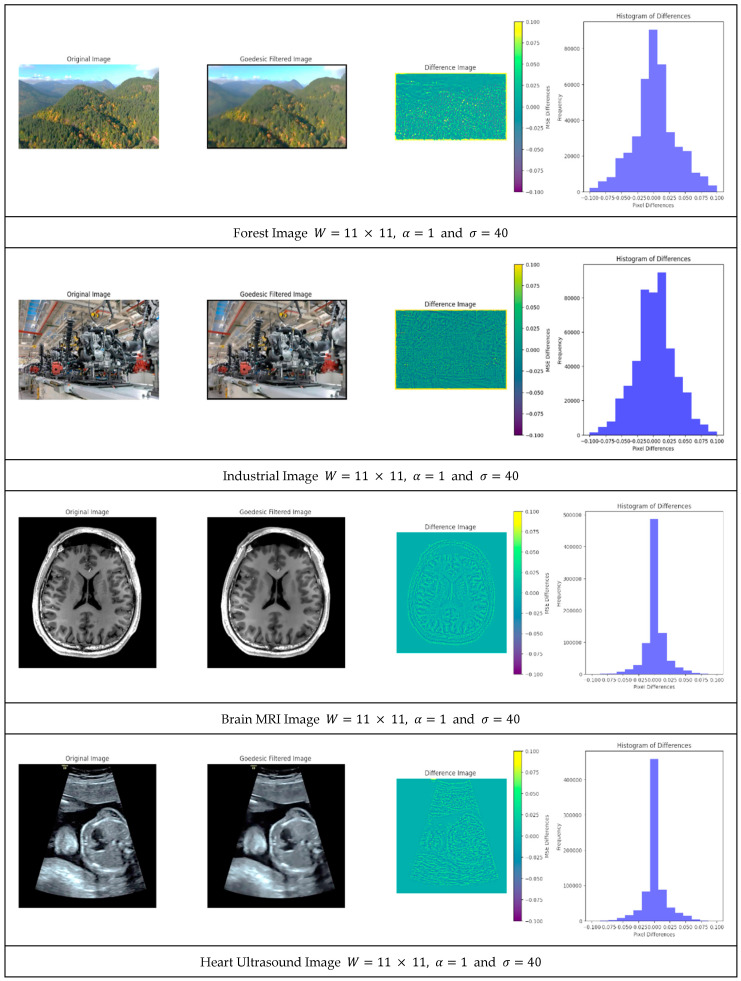
Difference between the original images and the filtered images.

The next experiment is to illustrate the distribution of the differences between the original image and the filtered one for a window size equal to 11 × 11 and a filter parameter equal to α = 1 and σ = 40. In Figure 7, one can see the original image, the corresponding filtered image, and the color-coded difference between the two images normalized between −0.1 and 0.1. In addition, one can see for each image a histogram of the error between −0.1 and +0.1.

The difference between the original image and the filtered images were very small, even though the filtered image was convolved heavily with σ=40. This sequence shows the advantage of geodesic filtering where uniform regions were smoothed out without losing sharp edges.

When an image is processed with geodesic filtering, the parameter σ controls the extent of the filtering effect. The “natural scale” represents the specific σ value that achieves the best balance between noise reduction and preservation of significant image features. This concept builds on scale-space theory, which states that images contain features at multiple scales. The natural scale has several key characteristics:

1. Peak Performance Point: As demonstrated in the paper, when plotting PSNR or SSIM values against increasing σ values, there is typically a clear peak before performance declines. This peak identifies the natural scale for that image.
2. Content Dependency: Each image has its own unique natural scale based on its content complexity. Images with fine textures typically have lower optimal σ values, while images with larger homogeneous regions have higher optimal σ values.
3. Feature Preservation Threshold: The natural scale represents the threshold at which the filtering process maximally preserves meaningful edges and structures while still effectively suppressing noise.
4. Adaptive Processing: Rather than applying a fixed σ value across all images, the concept of natural scale suggests that filtering should adapt to each image’s inherent structure.
5. Noise-Robust Analysis: When evaluating images with added noise, the natural scale remains relatively stable, showing that it is tied to the underlying image structure rather than noise characteristics.

We begin by establishing baseline performance with controlled noise conditions, then progressively introduce more challenging scenarios to demonstrate the robustness of geodesic filtering. To demonstrate this unique property of geodesic filter, let us study how the image evolves with σ for images that are corrupted by a Gaussian noise with an amplitude between [0, 35] and an average of 0. Following the foundational work of Zhang et al. [29], we performed a peak signal-to-noise ratio (PSNR) analysis for each image as a function σ. PSNR is defined as

(21) $$PSNR=10\;*\;{log}_{10}\left(\frac{{MAX}_{i}^{2}}{MSE}\right)$$

where ${MAX}_{i}$ is the maximum possible pixel value and MSE is the mean squared error.

In addition, for each σ, we also compute the structural similarity index measure (SSIM) as it provides a deeper insight into structural preservation. SSIM incorporates three components:

- Luminance comparison;
- Contrast comparison;
- Structural correlation is an important criterion to measure edge preservation.
- SSIM is defined as

(22) $$SSIM\left(x,y\right)=\frac{\left(2\mu_{x}\mu_{y}+c_{1}\right)\left(2\sigma_{xy}+c_{2}\right)}{\left(\mu_{x}^{2}+\mu_{y}^{2}+c_{1}\right)\left(\sigma_{x}^{2}+\sigma_{y}^{2}+c_{2}\right)}$$

where

- $\mu_{x}$ is the pixel sample mean of x;
- $\mu_{y}$ is the pixel sample mean of y;
- $\sigma_{x}^{2}$ is the variance of x;
- $\sigma_{y}^{2}$ is the variance of y;
- $c_{1}=\left(k_{1}V\right)$, $c_{2}=\left(k_{2}V\right)$;
- V the dynamic range of the pixel-values (typically 255);
- $k_{1}=0.01$ and $k_{2}=0.03$ by default.

**Figure 8:**
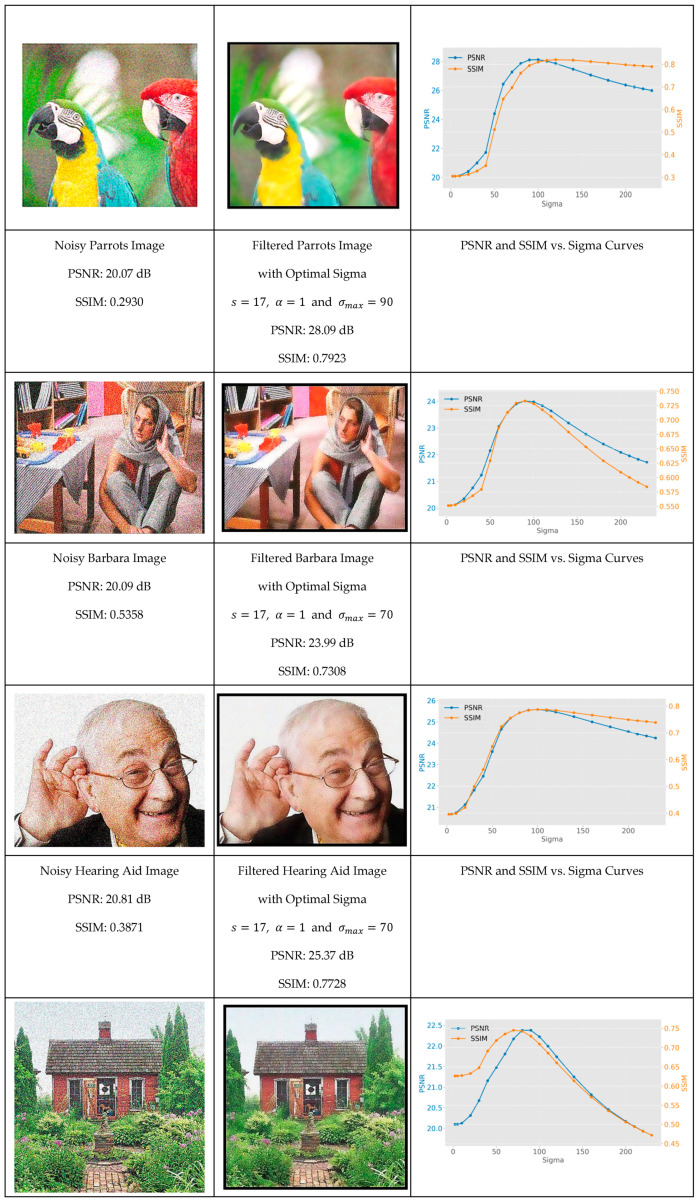

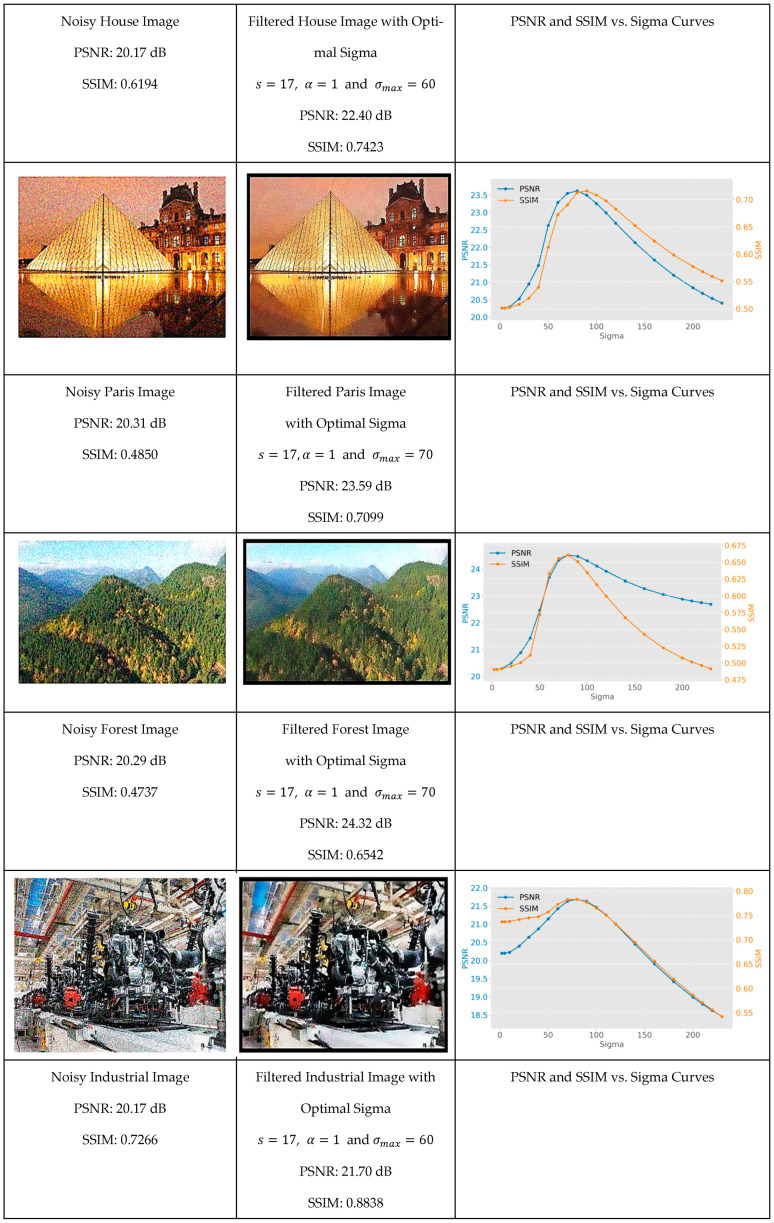

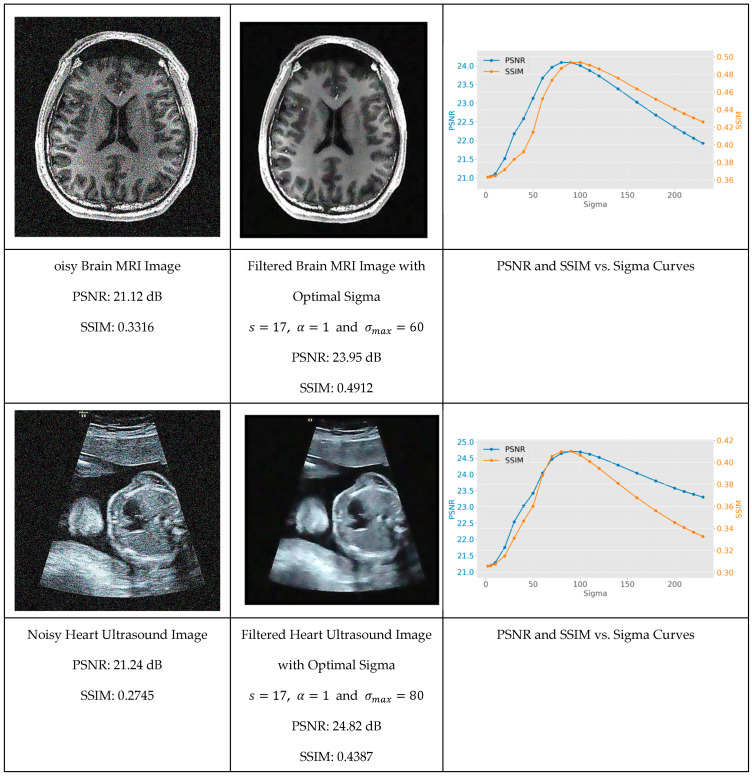
Evolution of the PSNR and SSIM as a function of σ for a noisy version of the dataset images. **Left**: The noisy image. **Center**: The image filtered with the optimum value $\sigma_{max}$. **Right**: The evolution of the PSNR and SSIM vs. σ.

Figure 8 shows the original image with Gaussian noise, the filtered version for $\sigma_{max}$ corresponding to the maximum of the PSNR, and finally the evolution of the PSNR and SSIM as a function of σ. One can see, for each image, the PSNR and SSIM evolve as a function of σ monotonically toward a maximum and then reduce due to over blurring. The $\sigma_{max}$ value corresponds to the maximum PSNR and is called the natural scale of the image.

The experimental results reveal that while the natural scale parameter varied across different images (with test cases showing a range from σ = 60 to σ = 90), it can be systematically identified through careful analysis of PSNR and SSIM metrics. This discovery provides researchers with a methodical framework for parameter optimization that eliminates the traditional trial-and-error approach commonly required in advanced filtering techniques.

### 4.3. Comparing Geodesic Filter to Other Algorithms

Previous studies comparing the performance of geodesic filtering and anisotropic diffusion methods have demonstrated the advantages and limitations of each approach. For instance, studies have shown that anisotropic diffusion methods, such as the Perona–Malik technique, are effective at preserving edges but struggle with highly textured or noisy images. While anisotropic diffusion methods have been widely adopted for their edge-preserving capabilities, geodesic filtering offers significant advantages in terms of noise reduction and spatial relationship preservation. A comparative study by Weickert [36] for grey-level images and by Boulanger [23] for range images underscore the potential of geodesic filtering as a superior technique for advanced image processing applications. Other studies by Gousseau et al. [37] compared various anisotropic diffusion methods and highlighted the potential of geodesic filtering in overcoming some of the inherent limitations of these techniques. Their findings suggest that geodesic filtering can provide better results in terms of both quantitative metrics, such as PSNR and SSIM by Gousseau et al. [37].

**Figure 9:**
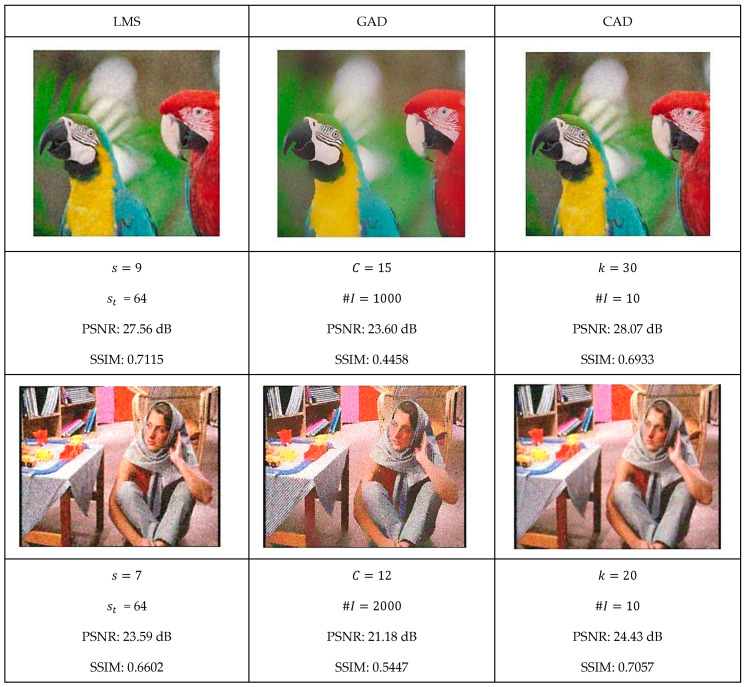

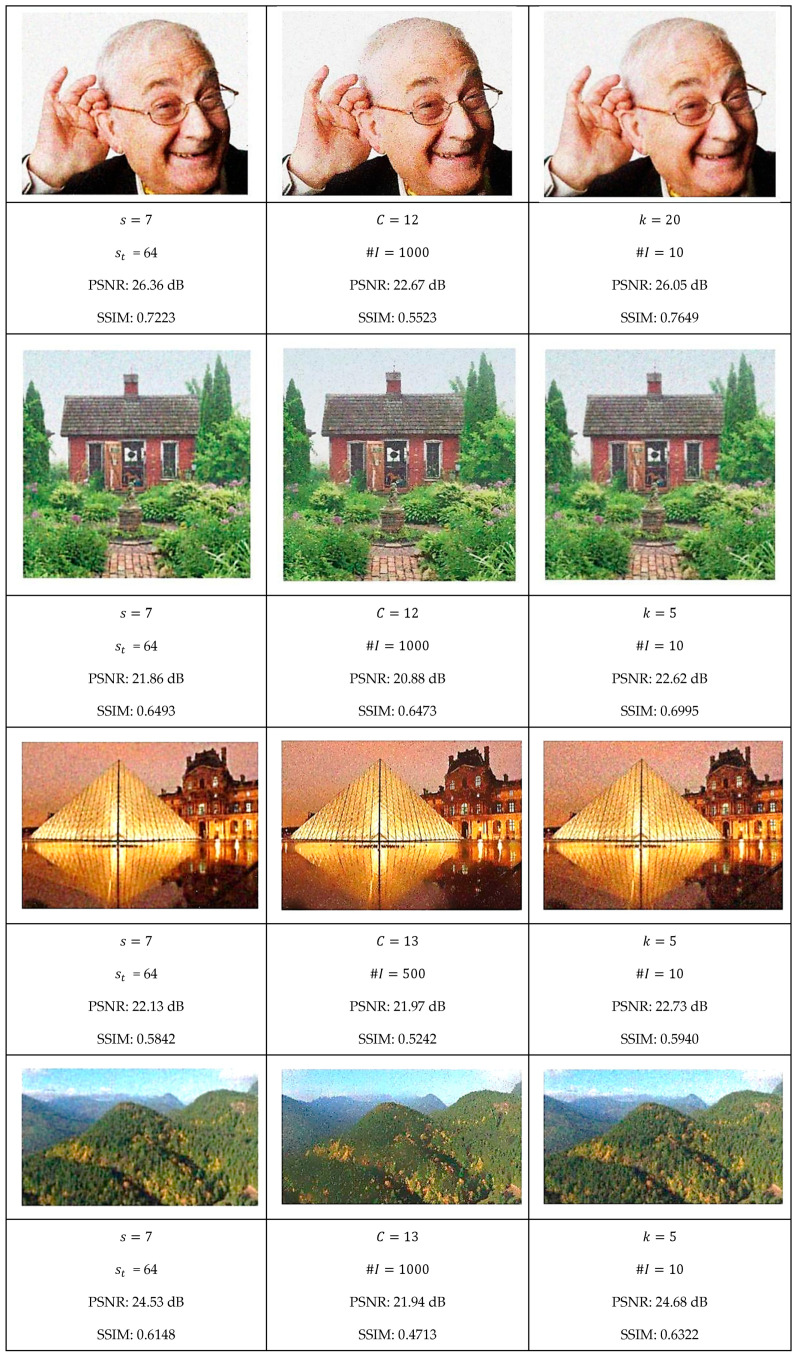

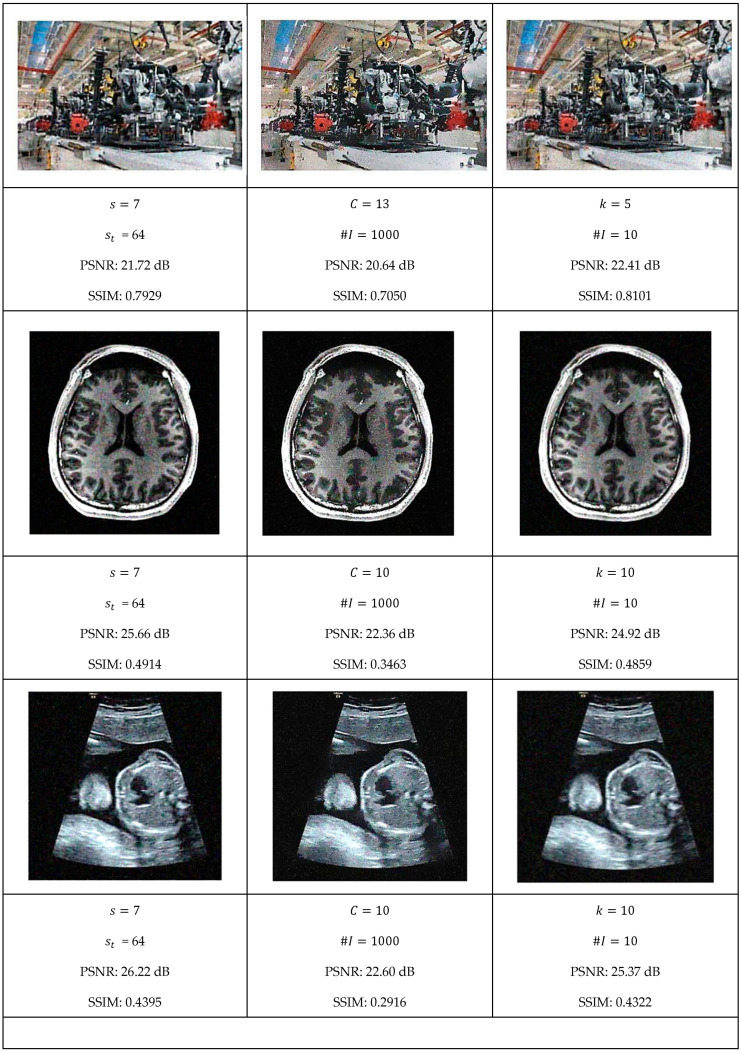
Optimal filtering results for LMS, gradient anisotropic diffusion, and curvature anisotropic algorithms.

**Figure 10:**
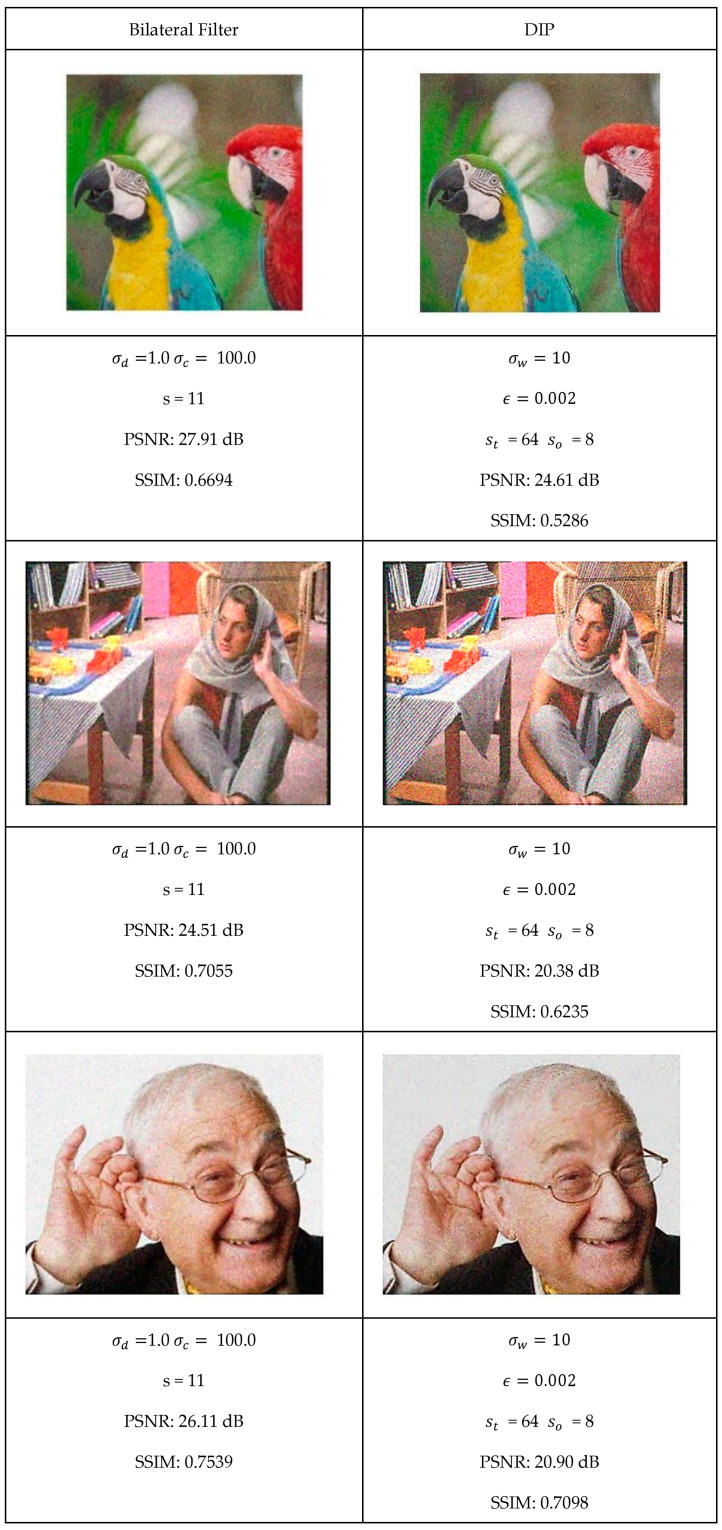

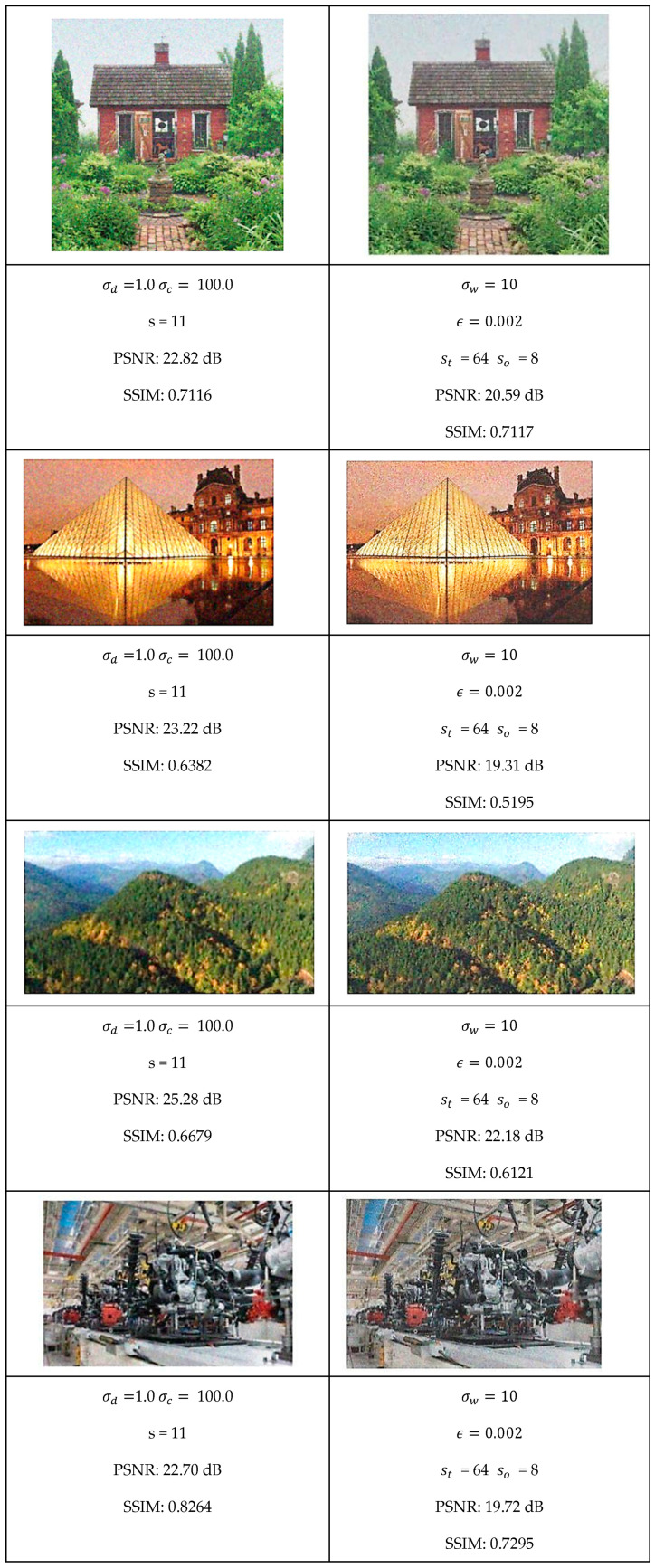

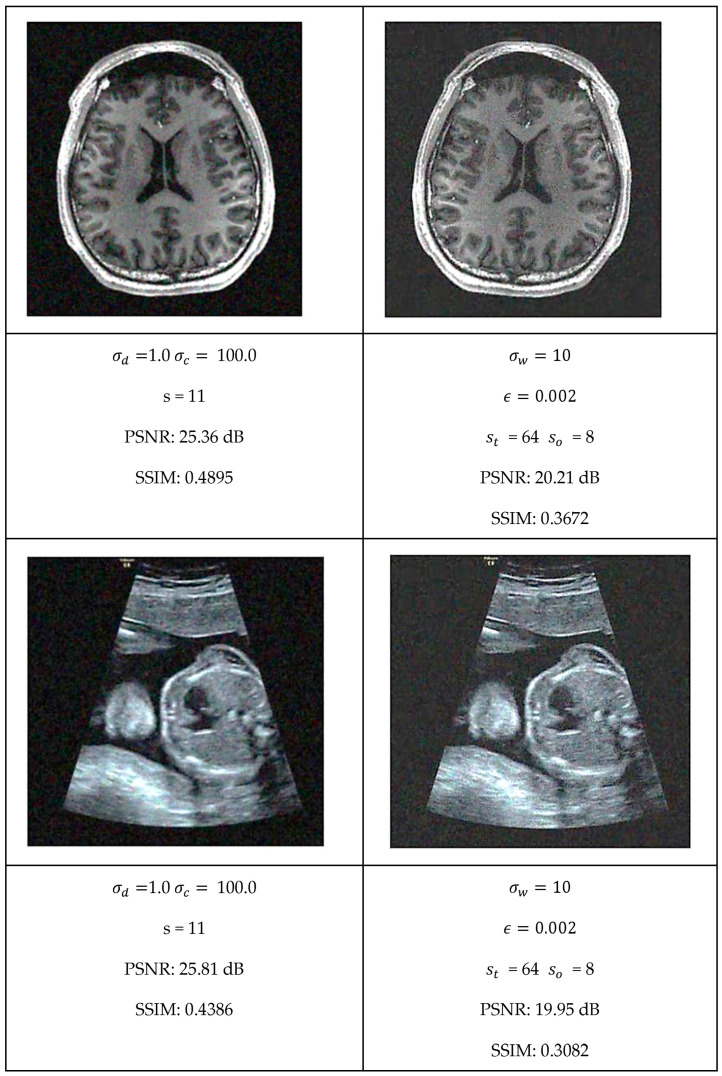
Optimal filtering results for bilateral and GW-DIP algorithms.

This section presents a comprehensive comparative analysis of our geodesic filtering approach against the state-of-the-art methods. The key to this comparison is based on PSNR and SSIM difference metrics. The noisy images shown in Figure 8 were processed using various implementations of the filtering algorithms described in Section 2. To be fair in our comparison, as with the geodesic filter, we tuned the parameters to produce the best PSNR value possible. The results of this comparison are collected in Figure 9 and Figure 10.

Each algorithm was carefully tuned to achieve optimal performance using the same test image database with standardized noise conditions. For each filter, the tuning parameters are as follows:

- Least Median Filter: window size s and tile size $s_{t}$;
- Gradient Anisotropic Diffusion: conductance C and number of iterations #I;
- Curvature Anisotropic Diffusion: the mean curvature of the level sets k and number of iterations #I;
- Bilateral Filter: s kernel size, $\sigma_{d}$ spatial distance weight, and $\sigma_{c}$ color distance weight;
- Gaussian Weighted Wavelet DIP Neural Network: $\sigma_{w}$ Gaussian variance, ϵ minimum tile loss, $s_{t}$ tile size, and $s_{o}$ tile overlap size.

In summary, the quantitative evaluation presented in Table 1 and Table 2 confirms that geodesic filtering provides dual performance advantages: achieving noise reduction metrics (PSNR) comparable to state-of-the-art alternatives while significantly outperforming them in structural preservation (SSIM). This superiority in preserving image structure is evident across the entire image dataset, with particularly notable advantages in regions containing intricate textures and fine details that traditional methods frequently over-smooth or distort.

The results clearly demonstrate that while other filtering approaches may achieve similar noise reduction performance, they do so at the cost of structural integrity. Geodesic filtering, in contrast, maintains the delicate balance between noise suppression and feature preservation, making it particularly valuable for applications where preserving the semantic content of images is paramount.

Furthermore, geodesic filtering shows remarkable resilience when processing non-standard noise distributions, including speckle patterns and mixed noise profiles that typically challenge conventional filters. This versatility extends its practical utility across diverse application domains from medical imaging to remote sensing.

## 5. Geodesic Filtering Computational Complexity and GPU Implementation

The computational complexity of geodesic filtering represents one of its most significant challenges, which necessitates the GPU implementation described in the paper. This aspect deserves thorough examination as it directly impacts the algorithm’s practical applicability. The computational bottleneck occurs specifically during the minimal path calculation (geodesic distance) between pixels. For each central pixel, the algorithm must compute the shortest path to every other pixel in the window, considering both spatial proximity and intensity differences. This requires solving multiple single-source shortest path problems, which have a complexity of O ($M\cdot Ns^{2}$) per window when using Dijkstra’s algorithm with a binary heap.

Like the algorithms described in Asad et al. [38] and Áfra et al. [39], our GPU implementation addresses many computational challenges through several innovative approaches:

1. Parallelization Strategy: By processing multiple image tiles simultaneously, the GPU implementation exploits the inherent parallelism in the algorithm. Each thread processes a single pixel, allowing thousands of pixels to be processed concurrently.
2. Memory Optimization: The paper describes sophisticated memory access patterns and bank conflict resolution techniques that significantly improve throughput on GPU architectures.
3. Wave Propagation Algorithm: The fast-marching method (FMM) implemented on GPU provides a more memory-access-friendly alternative to Dijkstra’s algorithm, eliminating the need for priority queues which cause memory bank conflicts.
4. Coalesced Memory Access: By arranging data in memory to enable coalesced access patterns, the implementation achieves near-optimal memory bandwidth utilization.

### 5.1. Memory Optimization

1. Image Tiling: A large image is divided into 16 × 16 tiles for processing, where each tile is handled by one thread block. A 7 × 7 convolution window requires an overlap of three pixels on each side (7/2⌋ = 3), resulting in an effective area processed by each tile to be 10 × 10 pixels (16-3-3).
2. Memory Layout: The input image is stored as RGB values (three channels) in global memory, where each pixel requires three float values. The image data are aligned for faster coalesced memory access from the threads. For each block, the threads read the data from global memory into the shared memory. Since each tile is 16 × 16, each block will read a tile of size 22 × 22 pixels (16 + 3 + 3 for each dimension) of three floats. This also includes halo regions. In addition, additional space is used in shared memory for distance computations.

#### 5.1.1. Block Execution Flow

1. Tile Loading Phase: Each 16 × 16 thread block loads the tile data (16 × 16), the halo region (three pixels each side), using coalesced reading from global memory.
2. Convolution Processing: Each thread is responsible for one pixel in 16 × 16 tiles, and threads near edges handle the halo region. First, a distance array is initialized. Each output pixel requires 7 × 7 window computation. The center pixel of each window is determined by thread ID. For a thread in the block,
3. The convolution window is centered at the current thread position;
4. Each thread processes the 7 × 7 window around center, which include
5. Computing geodesic distances within the window using the wave propagation algorithm;
6. Calculating the weight of the spatial and signal contributions using a Gaussian function;
7. Normalize the weights between zero and one;
8. Multiply the signal with the weights and the pixel by the weighted sum.

#### 5.1.2. Memory Access Optimization

The main optimization strategy is based on better use of memory access. First is a coalesced loading strategy where each thread loads one RGB pixel set 128-byte aligned access and where sequential thread IDs are mapped to sequential memory. For example, data are stored in global memory as [R_0_] [G_0_] [B_0_] [R_1_] [G_1_] [B_1_] … [R_15_] [G_15_] [B_15_]. Each thread in a half-warp read the data as follows:

- T0 → P0, P16, P32 (loads three pixels);
- T1 → P1, P17, P33;
- T2 → P2, P18, P34;
- …;
- T15 → P15, P31, P47.

The second data load consists of

- Loading halo region corresponding to an additional three pixels on each side;
- Maintain a coalesced pattern where possible;
- Handle boundary conditions near the edge of the image.

All the data for a tile of (22 × 22) are then stored into much faster shared memory as follows:

- [Halo] [Main Tile] [Halo];
- 3 + 16 + 3 = 22 columns;
- 3 + 16 + 3 = 22 rows.

It is stored as follows:

- [H H H|M M M M M M M M M M M M M M M M|H H H];
- [H H H|M M M M M M M M M M M M M M M M|H H H];
- [H H H|M M M M M M M M M M M M M M M M|H H H].

The main objective is to minimize memory bank conflict between threads in a block. Bank conflicts can cause 32× slowdown, as each conflict serializes access and also affects warp execution efficiency. First, we must ensure that each thread accesses different banks bank (T_xy_) ≠ bank (T_x’y’_) for any threads in the same warp. In our implementation, we used a column padding strategy where we added an extra column to the array to shift row starting addresses to ensure bank separation. Our implementation uses channel padding where we add an extra column to each channel:

- float r_channel [22,23];
- float g_channel [22,23];
- float b_channel [22,23].

This simplified bank mapping allows independent channel access that reduces conflict probability and better memory coalescing. This simple strategy eliminated bank conflicts to improve parallel memory operations of the full warp utilization. There is no serialization allowing for concurrent bank access and improved throughput.

### 5.2. Fast-Marching Method Algorithm (FMM)

The fast-marching method (FMM) represents a watershed advancement in geodesic distance computation, pioneered by Sethian’s [31] foundational work and subsequently refined for parallel architectures. At its core, FMM employs wavefront propagation—systematically expanding distance information outward from source points while maintaining strict causality principles essential for computational accuracy. This approach begins with a meticulous initialization phase that establishes the computational infrastructure through carefully configured distance maps and status markers for each vertex within the processing window. This preparatory stage, whose importance Peyré’s [27] research emphasized, creates the necessary foundation for subsequent processing steps. The algorithm’s defining characteristic lies in its disciplined processing sequence: examining points in strict order of increasing distance to ensure each vertex’s final distance value is definitively established before dependent calculations proceed. The causality-preserving ordering mechanism, whose mathematical properties Kimmel and Sethian [25] extensively analyzed, provide crucial guarantees regarding the precision of computed geodesic distances. This ordered processing framework enables FMM to efficiently resolve the Eikonal equation governing geodesic distance propagation, making it particularly amenable to GPU implementation through its structured memory access patterns and predictable data dependencies—characteristics that dramatically contrast with Dijkstra’s algorithm’s reliance on priority queues.

**Figure 11:**
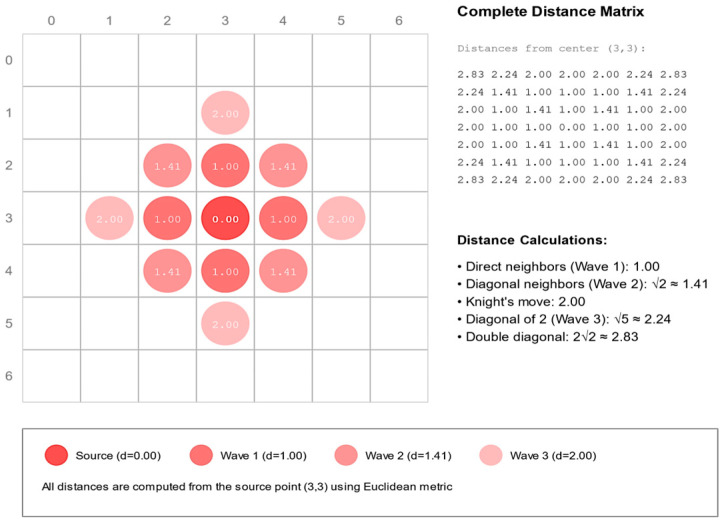
Wave front calculation for a 7 × 7 window.

The FMM algorithm consists of several key components:

1. Wave Structure: For our example, four concentric waves need to be processed by a thread. Each wave represents the front of pixels at similar geodesic distances that expand outward from a source point. Each wave elements are stored in shared memory contains position, distance, and color information.
2. Distance Update Mechanism: For each pixel in the current wave, the algorithm examines all 8-connected neighbors and then computes the geodesic distance combing spatial and color distances using Equation (14). The algorithm then updates the distance if a shorter path is found. All updates use atomic operators to avoid race conflicts between threads.
3. Wave Evolution: Initially, the wave inside a convolution window starts at the source point and then propagates outward. The wave size adapts based on local image properties. The process continues until all pixels in the window are reached. Each thread in a block examines its 8-connected neighbors and then computes a new distance if a shorter is found. Then, the neighbor is marked for inclusion in the next wave if necessary. See Figure 11 for an illustration of this process.

The FMM computational complexity $O\left(S\right)$ is less efficient than the Dijkstra algorithm. On the other hand, the memory access is more efficient for parallel implementation as it is not random.

### 5.3. Speed Comparison Between Python and CUDA Implementations

This GPU implementation-based wave propagation is more efficient and more adapted to GPU processing than the CPU implantation using Dijkstra’s algorithm. For Dijkstra’s algorithm for 7 × 7 windows:

- Nodes $\left(N\right)=49\;\left(7\times 7\right)$;
- Time Complexity: $O\left(N\;{log}_{2}\;N\right)$;
- For each pixel: 49 × ${log}_{2}\;$(49) operations to compute the geodesic distance;
- More calculation is also needed to maintain the priority queue.

For the wave propagation algorithm,

- We need to compute four waves (center, first ring, second ring, outer);
- Operations per wave: $O\left(N\right)$;
- Fixed number of steps, regardless of window content.

For a single 7 × 7 window, the Dijkstra algorithm requires

- Operations: ~280 (49 × ${log}_{2}$ (49);
- Forty-nine sequential steps;
- Random memory access due to the priority queue;
- Extra storage for the priority queue.

For the same window size, the wave propagation algorithm requires

- Operations: ~196 (49 × 4 waves);
- Structured memory access;
- Extra storage for the wave buffers.

### 5.4. Execution Speed Comparison

A comparison between a CPU implementation written in Python 3.8 and a GPU implementation written using CUDA Toolkit version CUDA 12.9 was performed. We used a powerful CPUs to compare with two GPUs running in the Google Colab environment. The CPU is the AMD Threadripper 7980X with the following specifications:

- Cores: 64;
- Threads: 128;
- Base Clock: 3.2 GHz;
- Boost Clock: 5.1 GHz;
- Memory Bandwidth: ~200 GB/s;
- L3 Cache: 384 MB.

The two GPUs used for the comparison were

1. the NVIDIA A100 GPU: CUDA Cores: 6912;
2. Memory: 80 GB HBM2e;
3. Memory Bandwidth: 2039 GB/s;
4. Base Clock: 1410 MHz.

and the

1. NVIDIA T4 GPU:
2. CUDA Cores: 2560;
3. Memory: 16 GB GDDR6;
4. Memory Bandwidth: 320 GB/s;
5. Base Clock: 585 MHz.

Performance metrics for 1024 × 1024 color image for the Python Dijkstra implementation running on the CPU:

- Pure Python: ~45,000 ms;
- NumPy/SciPy: ~12,000 ms;
- Numba JIT [*]: ~3000 ms;
- Numba Parallel [*]: ~800 ms.

For the CUDA C Wave Propagation version running on a NVIDIA A100 GPU:

- Processing: ~3 ms;
- Memory Transfer: ~1 ms;
- Total: ~4 ms;
- Speedup vs. Numba: ~200×.

For a NVIDIA T4 GPU:

- Processing: ~15 ms;
- Memory Transfer: ~3 ms;
- Total: ~18 ms;
- Speedup vs. Numba: ~44×.

The implementation achieves significant performance improvements through wave-specific optimizations, dynamic bank conflict resolution, and adaptive memory access patterns. Experimental results demonstrate a speedup of 200 times for the NVIDIA A100 GPU and 44 times for the NVIDIA T4 GPU compared to a multithread execution on the CPU using Numba Parallel.

### 5.5. Scalability Between Algorithms

Scalability is also good as illustrated by the following statistics. For the Python Dijkstra Processing using Numba Parallel, implementation execution time vs. image size are

- 512 × 512: ~200 ms;
- 1024 × 1024: ~800 ms;
- 2048 × 2048: ~3200 ms;
- Scale factor: ~4×.

For CUDA Wave Propagation (on a A100 NVIDIA GPU):

- 512 × 512: ~1 ms;
- 1024 × 1024: ~4 ms;
- 2048 × 2048: ~16 ms;
- Scale factor: ~4×.

CUDA Wave Propagation (on a T4 NVIDIA GPU):

- 512 × 512: ~4.5 ms;
- 1024 × 1024: ~18 ms;
- 2048 × 2048: ~72 ms;
- Scale factor: ~4×.

### 5.6. Accuracy Between CPU and GPU Versions

The CPU (Python Dijkstra) implementation uses 64-bit double precision by default and is our gold standard reference to compute geodesic distances. For the GPU Wave Propagation implementation, each CUDA core has a single precision accuracy meaning that the final distance relative error to the gold standard will be ~10⁻⁶. So, for our test images the average error are:

- CPU Dijkstra: 0.0 (reference);
- GPU Wave (A100): 3.1 × 10^−6^;
- GPU Wave (T4): 3.2 × 10^−6^.

## 6. Conclusions

This paper has presented a comprehensive analysis of geodesic filtering within the Riemannian framework for image processing, offering both a rigorous theoretical foundation and extensive experimental validation. Our investigation demonstrates that by modeling images as high-dimensional manifolds and computing minimal geodesic paths, this approach achieves remarkable improvements in noise reduction and edge preservation. Geodesic filtering delivers noise reduction on par with state-of-the-art alternatives measured using PSNR while significantly outperforming them in structural preservation measured by SSIM. The robust differential geometry underpinning geodesic filtering enables it to adeptly handle complex image structures, including challenging scenarios with varying noise characteristics and intricate edge patterns.

The experimental data clearly validate that GPU acceleration is essential for practical geodesic filtering, with NVIDIA A100 hardware delivering a 200× performance boost over optimized CPU implementations. This dramatic acceleration transforms geodesic filtering from a mere theoretical concept into a viable processing technique for real-world applications. By reducing computational barriers, the GPU implementation enables geodesic filtering to be effectively applied to large-scale datasets and time-sensitive processing requirements across medical imaging, remote sensing, and video processing domains.

Furthermore, our analysis reveals that the geometric approach to image filtering offers fundamental advantages beyond performance metrics alone. By respecting the intrinsic geometry of image content, geodesic filtering preserves semantic information that is often lost in traditional filtering approaches. The preservation of fine structural details, particularly along complex curved boundaries and in textured regions, maintains the diagnostic or analytical value of processed images—a critical consideration in domains where visual information directly informs decision-making processes. The adaptive nature of geodesic distances, which automatically adjust to local image characteristics, provides a self-regulating mechanism that reduces the need for parameters tuning across diverse image types. This adaptability proves especially valuable when processing heterogeneous datasets with varying noise profiles and structural complexities. Our experiments with clinical medical images, satellite imagery, and natural photographs demonstrate this versatility, with consistent performance advantages observed across these diverse domains.

Additionally, the theoretical framework established in this paper opens new avenues for further research, including potential extensions to temporal filtering for video sequences, integration with deep learning architectures as a geometrically informed processing layer, and application to higher-dimensional volumetric data common in modern medical imaging. The mathematical formalism of Riemannian geometry provides a robust foundation for these future developments, suggesting that geodesic filtering represents not just an incremental improvement but a fundamentally different paradigm for approaching image processing challenges.

## 7. Future Research Directions

Looking forward, several promising avenues exist to further enhance and expand the capabilities of geodesic filtering. A primary focus will be on reducing its computational complexity while maintaining its inherent advantages. Advances in parallel computing—such as multi-GPU architectures and emerging hardware accelerators—paired with algorithmic improvements inspired by anisotropic fast marching methods, may deliver the necessary efficiency gains.

Another exciting direction involves extending the geodesic filtering framework to higher-dimensional data. While our current work concentrates on two-dimensional images, early studies indicate that the approach can be naturally generalized to 3D volumetric datasets—such as CT or MRI scans—by mapping from R^3^ to R^4^ (e.g., u, v, w to x, y, z, I). Similarly, the method could be adapted for video processing by extending the mapping to higher dimensions (e.g., R^3^ to R^6^ for video sequences, encompassing spatial and temporal dimensions along with color information) and even 4D time-varying datasets (e.g., dynamic CT or ultrasound data).

These extensions will not only widen the applicability of geodesic filtering but also create new research opportunities in the analysis and visualization of complex, high-dimensional data. Many of these extensions of the geodesic filtering approach have been successfully tested in our laboratory and will be published in the near future.

## Figures and Tables

**Table 1:** Comparison of the PSNR for each filtering algorithm.

Image	Noisy Image	Geodesic	LMS	GAD	CAD	Bilaterial	DIP
Parrot	20.07 dB	28.09 dB	27.56 dB	23.60 dB	28.07 dB	27.91 dB	24.61 dB
Barbara	20.09 dB	23.99 dB	23.59 dB	21.18 dB	24.43 dB	24.51 dB	20.38 dB
Hearing Aid	20.81 dB	25.37 dB	26.36 dB	22.67 dB	26.05 dB	26.11 dB	20.90 dB
House	20.17 dB	22.40 dB	21.86 dB	20.88 dB	22.62 dB	22.82 dB	20.59 dB
Paris	20.31 dB	23.59 dB	22.13 dB	21.97 dB	22.73 dB	23.22 dB	19.31 dB
Forest	20.29 dB	24.32 dB	24.53 dB	21.94 dB	24.68 dB	25.28 dB	22.18 dB
Industrial	20.17 dB	21.70 dB	21.72 dB	20.64 dB	22.41 dB	22.70 dB	19.72 dB
Brain MRI	21.12 dB	23.95 dB	25.66 dB	22.36 dB	24.92 dB	25.36 dB	20.21 dB
Ultrasound	21.24 dB	24.82 dB	26.22 dB	22.60 dB	25.37 dB	25.81 dB	19.95 dB
**Average PSNR**	20.47 dB	24.25 dB	24.40 dB	21.98 dB	24.59 dB	24.85 dB	20.87 dB

**Table 2:** Comparison of the SSIM for each filtering algorithm.

Image	Noisy Image	Geodesic	LMS	GAD	CAD	Bilaterial	DIP
Parrot	0.2930	0.7923	0.7115	0.4458	0.6933	0.6694	0.5286
Barbara	0.5358	0.7308	0.6602	0.5447	0.7057	0.7055	0.6235
Hearing Aid	0.3871	0.7728	0.7223	0.5523	0.7649	0.7539	0.7098
House	0.6194	0.7423	0.6493	0.6473	0.6995	0.7116	0.7117
Paris	0.4850	0.7099	0.5842	0.5242	0.5940	0.6382	0.5195
Forest	0.4737	0.6542	0.6148	0.4713	0.6322	0.6679	0.6121
Industrial	0.7266	0.8838	0.7929	0.7050	0.8101	0.8264	0.7295
Brain MRI	0.3316	0.4912	0.4914	0.3463	0.4859	0.4895	0.3672
Ultrasound	0.2745	0.4387	0.4395	0.2916	0.4322	0.4386	0.3082
**Average SSIM**	0.4585	0.6907	0.6296	0.5032	0.6464	0.6556	0.5678

## Data Availability

The raw data supporting the conclusions of this article will be made available by the authors on request.
